# Exotic charge-density waves and superconductivity on the kagome lattice

**DOI:** 10.1093/nsr/nwaf414

**Published:** 2025-09-29

**Authors:** Ruiqing Fu, Jun Zhan, Matteo Dürrnagel, Hendrik Hohmann, Ronny Thomale, Jiangping Hu, Ziqiang Wang, Sen Zhou, Xianxin Wu

**Affiliations:** Institute of Theoretical Physics, Chinese Academy of Sciences, Beijing 100190, China; School of Physical Sciences, University of Chinese Academy of Sciences, Beijing 100049, China; School of Physical Sciences, University of Chinese Academy of Sciences, Beijing 100049, China; Institute of Physics, Chinese Academy of Sciences, Beijing 100190, China; Institut für Theoretische Physik und Astrophysik, Universität Würzburg, Am Hubland Campus Süd, Würzburg 97074, Germany; Institute for Theoretical Physics, ETH Zürich, 8093 Zürich, Switzerland; Institut für Theoretische Physik und Astrophysik, Universität Würzburg, Am Hubland Campus Süd, Würzburg 97074, Germany; Institut für Theoretische Physik und Astrophysik, Universität Würzburg, Am Hubland Campus Süd, Würzburg 97074, Germany; Institute of Physics, Chinese Academy of Sciences, Beijing 100190, China; New Cornerstone Science Laboratory, Beijing 100190, China; Department of Physics, Boston College, Massachusetts 02467, USA; Institute of Theoretical Physics, Chinese Academy of Sciences, Beijing 100190, China; School of Physical Sciences, University of Chinese Academy of Sciences, Beijing 100049, China; CAS Center for Excellence in Topological Quantum Computation, University of Chinese Academy of Sciences, Beijing 100049, China; Institute of Theoretical Physics, Chinese Academy of Sciences, Beijing 100190, China

**Keywords:** kagome lattice, loop-current order, random phase approximation, pairing symmetry

## Abstract

Loop current order has long been pursued in various electronic systems, including cuprates and honeycomb lattice materials, but its realization remains elusive in both experiment and theory. Intriguingly, recent experimental evidence for AV$_3$Sb$_5$ (A = K, Rb, Cs) and related kagome metals hints at the formation of orbital currents in the charge-density wave-ordered regime, providing a mechanism for spontaneous time-reversal symmetry breaking in the absence of local moments. However, concrete theoretical model realizations of the loop-current order in the kagome lattice have been very challenging and remain an outstanding, unresolved problem. In this work, we comprehensively explore the competitive charge instabilities of the spinless kagome lattice with inter-site Coulomb interactions at the pure-sublattice van Hove filling. From the analysis of the charge susceptibility, we find that, at the nesting vectors, while the onsite charge order is dramatically suppressed, the bond charge orders are substantially enhanced owing to the sublattice texture on the hexagonal Fermi surface. Furthermore, we demonstrate that nearest-neighbor and next-nearest-neighbor bonds are characterized by significant intrinsic real and imaginary bond fluctuations, respectively. The $2\times 2$ loop-current order is thus favored by the next-nearest-neighbor Coulomb repulsion. Interestingly, increasing interactions further leads to a nematic state with intra-cell sublattice density modulation that breaks the $C_6$ rotational symmetry. We further explore superconducting orders arising from onsite and bond charge fluctuations, and discuss our model’s implications for the experimental status quo.

## Teaser

Researchers reveal that electronic Coulomb interactions can produce an exotic correlated quantum matter with circulating current order that breaks the time-reversal symmetry spontaneously on the kagome lattice.

## INTRODUCTION

Exploring novel quantum states has been a central theme in contemporary condensed matter physics. One long-sought-after state is the exotic staggered flux phase, proposed from different perspectives in the same year: for strongly correlated systems on a square lattice [1] and for topological phases of matter on a honeycomb lattice [2]. In hole-doped cuprates, the staggered flux phase is associated with a staggered electric current order on the square lattice [3–5], which is closely related to the intra-unit-cell copper-oxygen loop-current order [6] and the *d*-density wave order [7]. This order is a charge bond order with imaginary order parameters and features looped current patterns along the bonds in lattices. These proposed exotic phases of matter spontaneously break the time-reversal symmetry (TRS) due to orbital circulating currents without spin-related magnetism. When the lattice translational symmetry is additionally broken [3–5,7], they describe the highly unconventional TRS-breaking loop-current charge-density wave (CDW) states. The loop-current order was suggested as a hidden order in the pseudogap phase in underdoped cuprates [7–9] and loop-current fluctuations have been shown to provide *d*-wave pairing instability [3,6]. In the Haldane model for spinless fermions on the honeycomb lattice [2], this flux phase breaks the time-reversal symmetry and leads to a quantum anomalous Hall (QAH) insulator, realizing the quantum Hall effect without Landau levels. It has become a prototype example for exploring topological phases of matter [10,11].

Despite its physical significance, concrete theoretical model realizations of loop-current order in the ground state have been very challenging. In the limit of strong electron-electron correlation, achieving loop-current order beyond the mean-field level remains controversial, partly due to partially conflicting many-body numerical evidence from the analysis of finite size systems [12–18]. While the induced orbital magnetism has uniquely testable experimental signatures, the experimental status of loop currents in the strongly correlated cuprates, in particular whether they live up to energy scales relevant to high-$T_c$ superconductivity, is still heavily debated [19,20]. For the honeycomb lattice at Dirac filling, the next-nearest-neighbor repulsion was suggested to promote a loop-current order, realizing an interaction-driven topological Haldane model [21]. However, unbiased numerical calculations, such as exact diagonalization, density-matrix renormalization group and functional renormalization group studies [22–26], reveal that the true ground state is a charge-ordered state with trivial topology. Additionally, while the quadratic band-touching point was proposed to generate a QAH state from the weak-coupling limit [27], its realization in the lattice model requires intricate interaction settings [28,29]. In both square and honeycomb lattice systems, the loop-current bond order always faces strong competition from onsite orders either in the charge or spin channel, making it less dominant. Therefore, devising a solid microscopic foundation for such a state beyond mean-field theory and biased variational methods has been particularly challenging.

The recent discoveries of kagome metals AV$_3$Sb$_5$ (A = K, Rb, Cs) [30–32] and FeGe [33–35], with the Fermi level close to van Hove singularities (VHSs) [34–37], offer new opportunities and fresh new ideas to explore this important issue on the geometrically frustrated kagome lattice. In non-magnetic AV$_3$Sb$_5$, a CDW order with translational symmetry breaking (TSB) occurs [38,39] and exhibits signatures of TRS from various experimental measurements [40–47]. In the kagome magnet FeGe, where magnetic moments align ferromagnetically within each layer and antiferromagnetically between layers, the emergence of CDW with TSB induces an enhancement in the magnetic moment and anomalous Hall effect [34]. This evidence implies the potential presence of loop-current order in kagome metals with a signal strength and data diversity far beyond existing evidence in cuprates [48–53]. The Fermi surface nesting vector associated with these VHSs is consistent with the in-plane wave vector of the CDW, implying their crucial role in promoting the CDW.

The VHSs in the kagome lattice exhibit a unique sublattice texture, leading to matrix element reduction effects of scattering channels between van Hove (VH) points, dubbed as sublattice interference (SI) [54,55]. Whenever the accumulation of electronic density of states at the VH points represents a relevant contribution to the formation of Fermi surface instabilities, it is to be expected that SI could have a crucial impact on the nature of the electronic order. Despite intensive theoretical studies, the question of whether electronic interactions within the kagome lattice, intertwined with SI, can give rise to loop-current states at VH filling remains an open issue. Studies using the functional renormalization group approach applied to the t-U-V model have not identified the presence of such orders [54,56–60]. Mean-field analyses, however, suggest that longer-range interactions, in particular the next-nearest-neighbor interaction, could have a crucial impact on the loop-current order [61]. Additionally, the topological loop-current order can be promoted by bond order fluctuations [62,63]. Few physical insights into this order within the kagome lattice have been elucidated.

In this article, we seek to perform model building that aims at providing a microscopic foundation for the loop-current order. Our work is guided by the core idea that SI in kagome metals could set the stage for loop-current orders reachable through a full-scale many-body analysis beyond mean-field approximation and finite-size studies. As we delve into the intrinsic charge orders of the kagome lattice, we further reduce complexity by examining a single orbital spinless fermion model on the kagome lattice, similar to the Haldane model on the honeycomb lattice, in the presence of inter-site Coulomb interactions. To go beyond mean-field approximation that neglects charge fluctuations, we employ the random phase approximation (RPA) approach, which allows us to treat both onsite and bond order on equal footing. Our analysis of charge susceptibilities reveals that while the onsite charge order will be suppressed, the bond charge order gets enhanced owing to SI from the sublattice texture associated with the *p*-type VHS. Moreover, facilitated by the unique lattice’s geometry, we observe that the nearest-neighbor (NN) and next-NN (NNN) bonds exhibit pronounced intrinsic real and imaginary bond charge fluctuations, respectively. The emergence of a $2\times 2$ loop-current order CDW as the ground state is favored when the NNN repulsion is sufficiently strong, while dominant NN repulsion favors a $2\times 2$ trihexagonal (inverse star of David) charge bond order. Strong inter-site repulsion can stabilize a novel nematic state characterized by charge-density modulations coupled with bond charge modulations within the unit cell. We further explore the nature of triplet superconductivity away from VH filling descending from such exotic charge orders within our model, where *p*- and *f*-wave pairings emerge due to bond charge fluctuations. Finally, we discuss possible experimental implications and contemplate future quantitative theoretical studies that build upon the conceptual narrative outlined in our work.

## TIGHT-BINDING MODEL AND SUSCEPTIBILITIES OF ONSITE AND BOND CHARGE ORDERS

**Figure 1.**
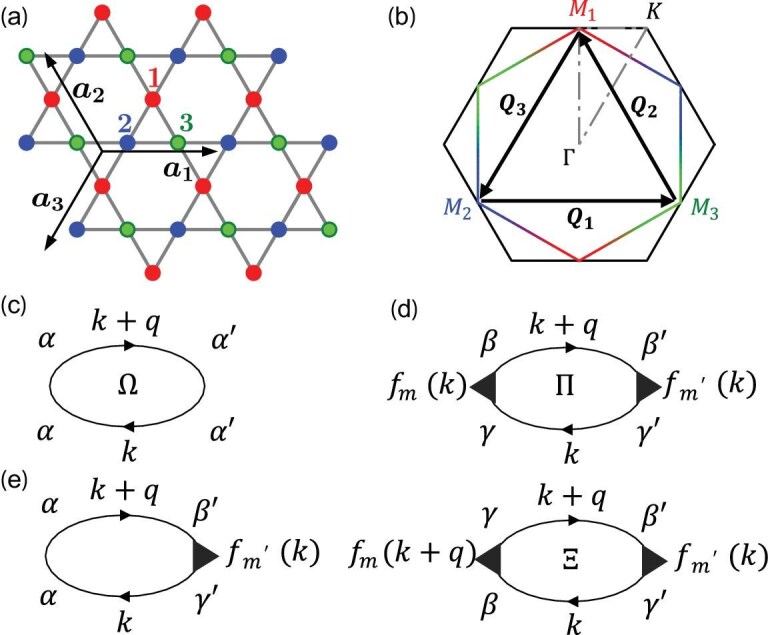
Kagome lattice and charge susceptibility bubbles. (a) Kagome lattice and (b) the sublattice-resolved Fermi surface at the *p*-type van Hove filling, with three sublattices indicated by red (1), blue (2) and green (3) circles. The two basis lattice vectors are ${{\boldsymbol a}}_1=(1,0)$, ${{\boldsymbol a}}_2=(-{1}/{2},{\sqrt{3}}/{2})$, with the third vector ${{\boldsymbol a}}_3=-{{\boldsymbol a}}_1-{{\boldsymbol a}}_2$. Feynman diagrams for susceptibilities in the onsite (c), bond (d) and mixed (e) channels. Here $\Omega$ is the onsite susceptibility and $\Pi$ and $\Xi$ are two bond susceptibilities, with $f_m$ (k) describing the corresponding form factors.

The kagome lattice consists of corner-sharing triangles with three sublattices, as shown in Fig. 1a. Analogous to the Haldane model, we consider the spinless kagome model and there are three motivations. First, as we are avoiding complicated magnetic orders via a frozen spin scenario, we can thoroughly study the effect of sublattice texture on charge fluctuations. Second, given the absence of magnetic phases for most kagome metals of our interest, the amount of competing density wave orders removed through this simplification is highly limited, and hence allows us to draw rather accurate implications for the spinful model. Third, the model is directly relevant to experiments, like antiferromagnet FeGe. The kinetic energy is described by the tight-binding Hamiltonian


(1) \begin{eqnarray*}
\mathcal {H}_0=-t\sum _{\langle {{\bf r}}{{\bf r}}^{\prime }\rangle ,\alpha \ne \beta } c^\dagger _{\alpha }({{\bf r}}) c_{\beta }({{\bf r}}^{\prime }) -\mu \sum _{{{\bf r}}, \alpha } n_{\alpha }({{\bf r}}),\\
\end{eqnarray*}

where $c^\dagger _{\alpha }({{\bf r}})$ and $c_{\alpha }({{\bf r}})$ are the creation and annihilation operators of an electron at the lattice site ${{\bf r}}$, $\alpha =1,2,3$ is the sublattice index, $\langle {{\bf r}}{{\bf r}}^{\prime }\rangle$ denotes the NN sites and $n_{\alpha }({{\bf r}})=c^\dagger _{\alpha }({{\bf r}}) c_{\alpha }({{\bf r}})$ is the electron density operator. Here *t* and $\mu$ are the NN hopping parameter and chemical potential, respectively. Defining *t* as the unit of energy, we set $t=1$ from now on. The tight-binding band structures feature two VHSs, one Dirac cone and a flat band. In particular, the Fermi surfaces at the two VH fillings are characterized by distinct sublattice textures [54,55]. In this work, we focus on the upper VH case at the pristine filling, i.e. the *p*-type VHS [54,55], and the sublattice-resolved Fermi surface is displayed in Fig. 1b. Clearly, the wave function at each saddle point (i.e. ${{{\bf M}}}$ point) is attributed to a single sublattice, while at the midpoint between two saddle points, the wave function exhibits an equal mixture of two sublattices. The Fermi surface nesting vectors ${{\bf Q}}_{1,2,3}$ always connect distinct sublattice characters of states around the three saddle points, leading to substantial bond fluctuations, as will be demonstrated in the subsequent analysis.

To examine the intrinsic fluctuations, we consider the relevant charge orders on the kagome lattice. The first one is charge modulation, i.e. the onsite charge order, and it is described by the operator $n_{\alpha }({{\bf r}})$ in real space. In momentum space, this operator reads


(2) \begin{eqnarray*}
n_{\alpha }({{\bf q}}) &=& \frac{1}{\sqrt{N}} \sum _{{{\bf r}}} e^{-i{{\bf q}}\cdot {{\bf r}}} n_{\alpha }({{\bf r}}) \\
&=& \frac{1}{\sqrt{N}} \sum _{{{\bf k}}} c^\dagger _{\alpha } ({{\bf k}}+{{\bf q}}) c_{\alpha }({{\bf k}}).
\end{eqnarray*}

We additionally consider bond charge modulation, i.e. charge bond order, on NN and NNN bonds. Because of the unique geometry of the kagome lattice, within each unit cell there are two NN (NNN) bonds along the direction parallel (perpendicular) to each basis vector ${{\boldsymbol a}}_\alpha$, and they all connect two distinct sublattices $\beta$ and $\gamma$, with the Levi-Civita symbol satisfying $\epsilon _{\alpha \beta \gamma }=1$, i.e. $(\alpha , \beta , \gamma )= (1, 2, 3), (2, 3, 1)$ and $(3, 1, 2)$. This allows us to define the symmetric ($+$) and antisymmetric ($-$) bond operators [52,61]


(3) \begin{eqnarray*}
B_{\alpha ,+,\eta } ({{\bf r}}) &=& \frac{1}{2} \Big[ c_\beta ^\dagger ({{\bf r}}) c_\gamma ({{\bf r}}+{{\bf l}}_{\alpha ,\eta }) \\
&&+ c_\beta ^\dagger ({{\bf r}}) c_\gamma ({{\bf r}}-{{\bf l}}_{\alpha ,\eta }) \Big],
\end{eqnarray*}

(4) \begin{eqnarray*}
B_{\alpha ,-,\eta } ({{\bf r}}) &=& \frac{i}{2} \Big[ c_\beta ^\dagger ({{\bf r}}) c_\gamma ({{\bf r}}+{{\bf l}}_{\alpha ,\eta }) \\
&&- c_\beta ^\dagger ({{\bf r}}) c_\gamma ({{\bf r}}-{{\bf l}}_{\alpha ,\eta }) \Big],
\end{eqnarray*}

where $\eta$ denotes NN and NNN bonds, and the corresponding displacement vectors connecting the two sites of the $\beta$ and $\gamma$ sublattices are ${{\bf l}}_{\alpha ,\text{NN}}=\frac{1}{2} {{\boldsymbol a}}_\alpha$ and ${{\bf l}}_{\alpha ,\text{NNN}}=\frac{1}{2} ({{\boldsymbol a}}_\beta - {{\boldsymbol a}}_\gamma \!)$, respectively. Performing Fourier transformation, the bond order operators in the momentum space can be written as


(5) \begin{eqnarray*}
B_{\alpha ,\pm ,\eta }({{\bf q}})=\frac{1}{\sqrt{N}} \sum _{{{\bf k}}} f_{\alpha ,\pm ,\eta } ({{\bf k}}) c^\dagger _{\beta }({{\bf k}}+{{\bf q}}) c_{\gamma }({{\bf k}})
\end{eqnarray*}

with $f_{\alpha ,+,\eta }({{\bf k}}) =\cos (\mathbf {k\cdot l}_{\alpha ,\eta })$ and $f_{\alpha ,-,\eta }({{\bf k}}) = \sin (\mathbf {k \cdot l}_{\alpha ,\eta })$ being the form factors of symmetric and antisymmetric bonds, respectively. We note that the antisymmetric bond defined in this work differs from that in [52] and [61] by a factor of *i*, which leads to a real form factor in Equation (5). Clearly, including NN and NNN bonds, there are in total 12 independent bond orders indexed by $(\alpha ,\pm ,\eta )$ within each unit cell. To simplify their indices, we introduce one-dimensional indices $m,n= \lbrace 1,2,\dots ,12\rbrace$  $=\lbrace ({1,+,\text{NN}})$, $({1,-,\text{NN}})$, $1\rightarrow 2,3$, $\text{NN}\rightarrow \text{NNN}\rbrace$. Note that the bond orders are complex in general; we thus introduce their conjugate partners as well: $B^\dagger _{\alpha ,\pm ,\eta }({{\bf r}}) \equiv [B_{\alpha ,\pm ,\eta }({{\bf r}})]^\dagger$ in real space and, consequently, $[B_{\alpha , \pm , \eta } ({{\bf q}})]^\dagger =B_{\alpha , \pm , \eta }^\dagger (-{{\bf q}})$ in momentum space.

To investigate the intrinsic fluctuations of different charge orders, we calculate the corresponding susceptibilities, defined as


(6) \begin{eqnarray*}
&&\chi _{pq} ({{\bf q}},i\omega _n) \\
&&\quad = \int _0^\beta d \tau e^{i\omega _n \tau } \langle T_\tau \mathcal {O}_p ({{\bf q}},\tau ) [\mathcal {O}_q ({{\bf q}},0)]^\dagger \rangle . \\
\end{eqnarray*}

Here operator $\mathcal {O}_p$ runs over the 27 charge orders mentioned above, consisting of 24 bond orders in the order of $\lbrace B_1, B^\dagger _1, B_2, B^\dagger _2, \dots , B_{12}, B^\dagger _{12}\rbrace$ followed by the three onsite charge orders $\lbrace n_1, n_2, n_3\rbrace$. The bare static susceptibility is given by $\chi ^0_{pq} ({{\bf q}})\equiv \chi _{pq} ({{\bf q}},0)$. For the convenience of discussion, we use different notation to distinguish the susceptibilities of onsite and bond charge orders in the following, and we further note that the latter can be categorized into two types. Explicitly, a $3\times 3$ susceptibility matrix for onsite charge orders $\Omega ^0_{\alpha \beta }$ = $\chi ^0_{24+\alpha , 24+\beta }$, and $24\times 24$ susceptibilities for bond charge orders $\Pi ^0_{mn}$ = $\chi ^0_{2m-1,2n-1}$ = $\chi ^0_{2m,2n}$ and $\Xi ^0_{mn}$ = $\chi ^0_{2m-1,2n}$ = $\chi ^0_{2m,2n-1}$ with $m,n=1,\dots ,12$ corresponding to the 12 independent bond order indices introduced in the above paragraph. The $\Omega$ operators represent onsite charge fluctuations, while the $\Pi$ and $\Xi$ operators represent bond fluctuations characterized by the correlation between $B/B^\dagger$ and $B^\dagger /B$, and between $B/B^\dagger$ and $B/B^\dagger$, respectively. The corresponding Feynman diagrams for $\Omega$ are just the normal bubbles, while those for $\Pi$ and $\Xi$ carry two additional vertices of form factors, as depicted in Fig. 1c and 1d. The analytical expressions are


(7) \begin{eqnarray*}
\Omega ^0_{\alpha \beta }({{\bf q}}) = -\frac{T}{N} \sum _{{{\bf k}},l} G^0_{\beta \alpha } ({{\bf k}}+{{\bf q}}, i\omega _l) G^0_{\alpha \beta } ({{\bf k}},i\omega _l),\\
\end{eqnarray*}

(8) \begin{eqnarray*}
\Pi ^0_{mn}({{\bf q}})&=& -\frac{T}{N} \sum _{{{\bf k}},l} f_m({{\bf k}}) f_n({{\bf k}})G^0_{\beta _n \beta _m} ({{\bf k}}+{{\bf q}}, i\omega _l) \\
&& \times G^0_{\gamma _m \gamma _n}({{\bf k}}, i\omega _l),
\end{eqnarray*}

(9) \begin{eqnarray*}
\Xi ^0_{mn} ({{\bf q}})&=& -\frac{T}{N} \sum _{{{\bf k}},l} f_m({{\bf k}}+{{\bf q}}) f_n({{\bf k}})G^0_{\beta _n \gamma _m} \\
&& \times({{\bf k}}+{{\bf q}}, i\omega _l) G^0_{\beta _m \gamma _n} ({{\bf k}},i\omega _l),
\end{eqnarray*}

where $G^0_{\beta \gamma } ({{\bf k}},i\omega _l)=\sum _\nu a_{\beta \nu } ({{\bf k}}) a^{*}_{\gamma \nu } ({{\bf k}}) /(i\omega _l-\epsilon _{\nu {{\bf k}}})$ is the noninteracting Green function with $\epsilon _{\nu {{\bf k}}}$ the $\nu$th eigenenergy of $\mathcal {H}_0$ and $a_{\beta \nu }({{\bf k}})$ the corresponding eigenstate. The summation over the fermionic Matsubara frequency $\omega _l=(2l+1)\pi k_BT$ at temperature *T* yields the Lindhard function and sublattice-associated matrix elements; for detailed formulas, see the online supplementary material. These sublattice characters embedded in the noninteracting Green functions play a predominant role in determining the behavior of susceptibilities. The diagram with only one vertex displayed in Fig. 1e represents the susceptibility in the mixed channel that couples the onsite and bond charge orders.

Before presenting the numerical data, we analyze the contributions to the bare susceptibilities from the VH points. At the *p*-type VH filling, the hexagonal Fermi surface encompasses the three inequivalent VH points labeled by ${{{\bf M}}}_{1,2,3}$ at the zone boundary and features perfect nesting with three wave vectors ${{\bf Q}}_\alpha = {1\over 2}{{\bf G}}_\alpha$, where ${{\bf G}}_\alpha$ denotes the reciprocal wave vector of the kagome lattice. These VH points with diverging density of states (DOS) are expected to contribute dominantly to the susceptibilities, especially at the two pertinent vectors, ${{\bf q}}=0$ and ${{\bf q}}= {{\bf Q}}_\alpha \equiv {{{\bf M}}}_\alpha$. Furthermore, as mentioned before and shown in Fig. 1b, the Bloch states at the ${{{\bf M}}}_\alpha$ points are exclusively localized on the $\alpha$th sublattice. Consequently, the Green functions at ${{{\bf M}}}_\alpha$ are nonvanishing only for $G^0_{\alpha \alpha }({{{\bf M}}}_\alpha )$. It is thus straightforward to show that these VH points contribute only to the diagonal elements of the onsite charge susceptibilities at ${{\bf q}}=0$, $\Omega _{\alpha \alpha }(0)$, while their contributions to off-diagonal elements of $\Omega (0)$ and all bond susceptibilities, $\Pi (0)$ and $\Xi (0)$, vanish. This results in a dominant onsite charge fluctuation at ${{\bf q}}=0$. The situation is, however, completely the opposite for wave vector ${{\bf q}}={{{\bf M}}}_\alpha$. The two VH points ${{{\bf M}}}_\beta$ and ${{{\bf M}}}_\gamma$ connected by ${{\bf q}}={{\bf Q}}_\alpha$ feature pure $\beta$th and $\gamma$th sublattices, respectively. This feature leads to the vanishing contribution of VH points in the onsite charge fluctuation $\Omega ^0_{\alpha ^{\prime }\alpha ^{\prime \prime }}({{{\bf M}}}_\alpha )$. But, these two VH points can thus dominantly contribute to the susceptibilities $\Pi ({{{\bf M}}}_\alpha )$ of the bonds that connect $\beta$ and $\gamma$ sublattices. We note that they have no contributions to $\Xi ({{{\bf M}}}_\alpha )$ since at least one of the two Green functions in Equation (9) involves mixed sublattices. This indicates predominant bond fluctuations at ${{\bf q}}={{{\bf M}}}_\alpha$ rather than onsite charge fluctuations. Furthermore, since ${{{\bf M}}}_{\beta /\gamma } \cdot {{\bf l}}_{\alpha ,\text{NN}} ={\pi /2}$ and ${{{\bf M}}}_{\beta /\gamma } \cdot {{\bf l}}_{\alpha ,\text{NNN}} =\pm {\pi /2}$, the contribution from these two VH points to the susceptibilities of symmetric bonds with form factors $\cos (\mathbf {k\cdot l}_{\alpha ,\eta })$ also vanishes. Therefore, at the wave vector ${{\bf q}}={{{\bf M}}}_\alpha$, the two connected VH points at ${{{\bf M}}}_\beta$ and ${{{\bf M}}}_\gamma$, with $\epsilon _{\alpha \beta \gamma }=1$, contribute only to the elements of $\Pi ({{{\bf M}}}_\alpha )$ associated with the antisymmetric bonds that connect $\beta$ and $\gamma$ sublattices. Explicitly, taking ${{\bf q}}={{{\bf M}}}_1$ as an example, the VH points contribute only to susceptibility $\Pi _{22}({{{\bf M}}}_1)$ for bond $B_2 =B_{1,-,\text{NN}}$, $\Pi _{88}({{{\bf M}}}_1)$ for bond $B_8=B_{1,-,\text{NNN}}$, and $\Pi _{28} ({{{\bf M}}}_1)$ that couples $B_2$ and $B_8$. Clearly, the unique sublattice texture at the *p*-type VH filling plays a pivotal role in suppressing the onsite charge fluctuations at wave vector ${{\bf q}}={{{\bf M}}}_\alpha$, but significantly promotes the bond charge fluctuations in the antisymmetric channel. This behavior in the kagome lattice markedly differs from what is observed in the triangular and honeycomb lattices, where onsite charge fluctuations are dominant [64,65]. Additionally, it is readily shown that the contribution from the VH points to the susceptibilities in the mixed channels depicted in Fig. 1e vanishes as well at both ${{\bf q}}=0$ and ${{\bf q}}={{{\bf M}}}_\alpha$.

**Figure 2.**
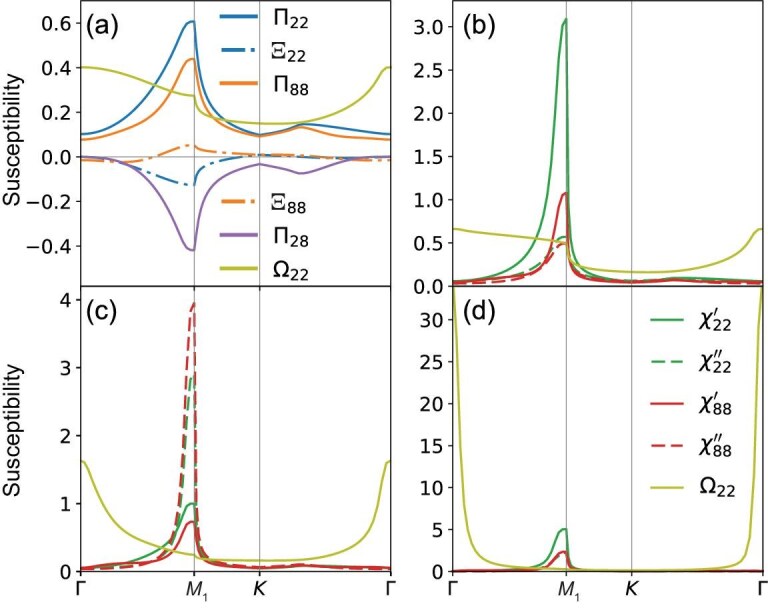
Bare and RPA susceptibilities along the high-symmetry path. (a) Representative components of the bare susceptibility for onsite and bond charge orders. Representative components of the RPA susceptibilities for onsite and bond charge orders at various inter-site Coulomb interactions: (b) $V_{\text{NN}}=0.6$, $V_{\text{NNN}}=0.0$, (c) $V_{\text{NN}}=0.0$, $V_{\text{NNN}}=0.95$ and (d) $V_{\text{NN}}=0.5$, $V_{\text{NNN}}=0.75$. We adopt $k_B T = 0.005$ in (a–c), while a higher temperature $k_B T = 0.01$ in (d) is used to avoid divergence. Explicit expressions of the susceptibilities shown in the legend are listed in abbreviated form: $\Pi _{22}=\langle B_{1,-,\text{NN}} B^\dagger _{1,-,\text{NN}} \rangle$, $\Xi _{22}=\langle B_{1,-,\text{NN}} B_{1,-,\text{NN}} \rangle$, $\Pi _{88}=\langle B_{1,-,\text{NNN}} B^\dagger _{1,-,\text{NNN}} \rangle$, $\Xi _{88}=\langle B_{1,-,\text{NNN}} B_{1,-,\text{NNN}} \rangle$, $\Pi _{28}=\langle B_{1,-,\text{NN}} B^\dagger _{1,-,\text{NNN}} \rangle$, $\Omega _{22}=\langle n_2 n_2 \rangle$.

The calculated bare susceptibilities are presented in detail in the online supplementary material, with the representative elements displayed in Fig. 2a along the high-symmetry path $\Gamma$-${{{\bf M}}}_1$-${\bf K}$-$\Gamma$ depicted in Fig. 1b. A temperature of $k_BT = 0.005$ is applied in the calculation, under which the dominant fluctuations are reflected by the peaks at ${{\bf q}}=0$ and ${{\bf q}}={{{\bf M}}}_\alpha$. Indeed, as suggested by the above analysis of the contribution from the VH points, the diagonal elements of onsite charge susceptibilities $\Omega _{\alpha \alpha }$ dominate at ${{\bf q}}=0$, while the leading susceptibilities at ${{\bf q}}={{{\bf M}}}_1$ are $\Pi _{22}$, $\Pi _{88}$ and $\Pi _{28}$ of the antisymmetric bonds connecting the two sites of the second and third sublattices. The large $\Pi ^0_{28}({{{\bf M}}}_1)$ shown in Fig. 2a indicates the strong coupling between the NN and NNN antisymmetric bond orders. These results promote the leading fluctuations in the antisymmetric bond channel, instead of symmetric bond or onsite charge channels. However, the nature of the antisymmetric bond order is yet to be explored.

To reveal the nature of the antisymmetric bond order that exhibits the leading fluctuation at the nesting wave vector ${{{\bf M}}}_\alpha$, we further separate the bond orders into their real and imaginary parts:


(10) \begin{eqnarray*}
B^{\prime }_m({{\bf q}}) &=& \frac{1}{2} \left[B_m + B^\dagger _m\right] \\
\text{and}\quad B^{\prime \prime }_m({{\bf q}}) &=& \frac{1}{2i} \left[B_m - B^\dagger _m \right], \quad m\in \text{odd},
\end{eqnarray*}

(11) \begin{eqnarray*}
B^{\prime }_m({{\bf q}}) &=& \frac{1}{2i} \left[B_m - B^\dagger _m \right] \\
\text{and}\quad B^{\prime \prime }_m({{\bf q}}) &=& \frac{1}{2} \left[B_m + B^\dagger _m \right], \quad m\in \text{even}.\\
\end{eqnarray*}

Here the definition in the antisymmetric bond channel is different because of the additional factor *i* used in Equation (4). By $B^{\prime }_m$ and $B^{\prime \prime }_m$ we represent the hopping and current modulation on the bonds, respectively. It is straightforward to show that the static susceptibilities of the real and imaginary bond charge orders can be respectively rewritten as


(12) \begin{eqnarray*}
&&{\chi ^{\prime }_{mm} ({{\bf q}}) = [\Pi _{mm}(\boldsymbol {q}) - (-1)^m \Xi _{mm}(\boldsymbol {q})]/2} ,\\
&&{\chi ^{\prime \prime }_{mm}(\boldsymbol {q}) = [\Pi _{mm}(\boldsymbol {q}) + (-1)^m \Xi _{mm}(\boldsymbol {q})]/2}.\\
\end{eqnarray*}

Clearly, the relative strength of real and imaginary bond fluctuations is dictated by the sign of $\Xi ({{{\bf M}}}_\alpha )$. According to the previous line of reasoning, the VH points contribute nothing to $\Xi$, rendering these fluctuations degenerate when considering only states at VH points. As a result, one has to go *beyond* the VH points and consider the contributions from other portions of the hexagonal Fermi surface (FS) to determine the sign of $\Xi ({{{\bf M}}}_\alpha )$.

We consider the diagonal elements of $\Xi$ at ${{\bf q}}={{{\bf M}}}_\alpha$ for the antisymmetric bonds connecting the $\beta$ and $\gamma$ sublattices, i.e. NN bond $B_{2\alpha }$ and NNN bond $B_{6+2\alpha }$, that are tied to the leading fluctuation. Because of the unique geometry of the kagome lattice, the nesting and connecting vectors satisfy ${{\bf l}}_{\alpha ,\text{NN}}\parallel {{\bf Q}}_{\alpha }$ and ${{\bf l}}_{\alpha ,\text{NNN}}\perp {{\bf Q}}_{\alpha }$, leading to ${{\bf Q}}_\alpha \cdot {{\bf l}}_{\alpha ,\text{NN}} =\pi$ and ${{\bf Q}}_\alpha \cdot {{\bf l}}_{\alpha ,\text{NNN}} =0$. Consequently, for ${{\bf k}}$ on the hexagonal FS, the two form factors in $\Xi _{2\alpha ,2\alpha }({{\bf Q}}_\alpha )$ for the NN antisymmetric bond read $f_{2\alpha }({{\bf k}}+{{\bf Q}}_\alpha ) f_{2\alpha }({{\bf k}})= -\sin ^2({{\bf k}}\cdot {{\bf l}}_{\alpha ,\text{NN}}) \le 0$, whereas the two form factors in $\Xi _{6+2\alpha ,6+2\alpha }({{\bf Q}}_\alpha )$ for the NNN antisymmetric bond are given by $f_{6+2\alpha }({{\bf k}}+{{\bf Q}}_\alpha ) f_{6+2\alpha }({{\bf k}})= \sin ^2({{\bf k}}\cdot {{\bf l}}_{\alpha ,\text{NNN}}) \ge 0$. These distinctive characteristics suggest that $\Xi _{2\alpha ,2\alpha } ({{\bf Q}}_\alpha )$ and $\Xi _{6+2\alpha ,6+2\alpha }({{\bf Q}}_\alpha )$ have opposite signs, pointing to the different nature of bond fluctuations on the NN and NNN bonds. Indeed, as shown in Fig. 1a, $\Xi _{22}$ is negative while $\Xi _{88}$ is positive at ${{\bf q}}={{{\bf M}}}_1$ due to the positive Green function–related sublattice factors. From Equation (12), it is apparent that, in the antisymmetric channel, the bare real bond fluctuation on the NN bonds is more pronounced, whereas the bare imaginary bond fluctuation is stronger on the NNN bonds. These distinctive characteristics, determined by the sublattice texture and unique geometry in the kagome lattice, open up the possibility of realizing exotic electronic orders, such as loop-current ground states.

## COMPETING ELECTRONIC STATES WITH INTER-SITE COULOMB INTERACTIONS

After studying the intrinsic charge fluctuations at the VH filling, we turn to explore the effect of electronic interactions on them. In the spinless scenario, the onsite Coulomb repulsion is absent by Pauli exclusion and we consider the NN and NNN inter-site Coulomb repulsions,


(13) \begin{eqnarray*}
\mathcal {H}_{\text{int}} &=& \sum _\eta V_\eta \sum _{\alpha , {{\bf r}}} [ n_\beta ({{\bf r}}) n_\gamma ({{\bf r}}+{{\bf l}}_\eta ) \\
&& +\, n_\beta ({{\bf r}}) n_\gamma ({{\bf r}}-{{\bf l}}_\eta ) ] \\
&=& \frac{1}{N} \sum _{\eta , \alpha } \sum _{{{\bf k}}, {{\bf k}}^{\prime }, {{\bf q}}} 2 V_\eta ({{\bf q}})c^\dagger _\beta ({{\bf k}}) c_\beta ({{\bf k}}+{{\bf q}}) \\
&& \times \, c^\dagger _\gamma ({{\bf k}}^{\prime }+{{\bf q}}) c_\gamma ({{\bf k}}^{\prime }) \\
\end{eqnarray*}

with $V_\eta ({{\bf q}})= V_\eta \cos ({{\bf q}}\cdot {{\bf l}}_{\alpha ,\eta })$. The interactions can be decoupled in terms of onsite charge orders


(14) \begin{eqnarray*}
\mathcal {H}_{\text{int}} = \sum _{\eta ,\alpha ,{{\bf q}}}2 V_\eta ({{\bf q}}) [n_\beta ({{\bf q}})]^\dagger n_\gamma ({{\bf q}})
\end{eqnarray*}

or offsite bond orders


(15) \begin{eqnarray*}
\mathcal {H}_{\rm {int}} = -\sum _{\eta ,\alpha ,{{\bf q}}} \sum _{s=\pm } 2 V_\eta [B_{\alpha ,s,\eta } ({{\bf q}})]^\dagger B_{\alpha ,s,\eta }({{\bf q}}).
\end{eqnarray*}

Once these interactions are introduced, both onsite and bond charge susceptibilities get renormalized. The first-order ladder of bond susceptibilities can be decomposed into the product of bond susceptibilities in different channels, as the interaction carrying internal momentum of fermion propagators can be decoupled owing to Equation (15), derived from $V_\eta ({{\bf k}}-{{\bf k}}^{\prime })=V_\eta \sum _{s=\pm } f_{\alpha ,s,\eta } ({{\bf k}}) f_{\alpha ,s,\eta } ({{\bf k}}^{\prime })$. The first-order bubble of bond susceptibilities will introduce a susceptibility in the mixed channel with only one vertex (as shown in Fig. 1e), which is the coupling between bond and onsite charge orders. Then, the first-order bubble of this mixed susceptibility will involve the onsite susceptibility. This hierarchy structure can be treated within the susceptibility matrix $\chi$, which involves both onsite and bond charge orders. We employ the RPA summation of all bubble and ladder diagrams (details in the online supplementary material), which yields the renormalized susceptibility matrix


(16) \begin{eqnarray*}
\chi _{\text{RPA}}({{\bf q}})=[1+\chi ^{0}({{\bf q}})\mathcal {U}_c(\boldsymbol {{{\bf q}}})]^{-1}\chi ^{0}({{\bf q}}).
\end{eqnarray*}

The interaction matrix ${\mathcal {U}_c({{\bf q}})}$ is given by


\begin{eqnarray*}
\mathcal {U}_c({{\bf q}}) = \left({\begin{array}{ccc} V^c_{\text{NN}}&\quad 0 &\quad 0 \\
0 &\quad V^c_{\text{NNN}} &\quad 0 \\
0 &\quad 0 &\quad U^c({{\bf q}}) \end{array}}\right),
\end{eqnarray*}

where


\begin{eqnarray*}
{U}^c({{\bf q}}) &=& \left({\begin{array}{ccc} 0 &\quad V_{12} &\quad V_{13}\\
V_{12} &\quad 0 &\quad V_{23} \\
V_{13} &\quad V_{23} &\quad 0 \end{array}}\right),\qquad\,\,\,\qquad(17) \\
V^c_{\eta } &=& -\text{diag}\lbrace 2V_{\eta },2V_{\eta },\dots \rbrace , \\
V_{\beta \gamma } &=& 2V_{\text{NN}}f_{\alpha ,+,\text{NN}}({{\bf q}})+2V_{\text{NNN}} f_{\alpha ,+,\text{NNN}}({{\bf q}}),
\end{eqnarray*}

with the indices $\alpha ,\beta ,\gamma$ being in $(\alpha ,\beta ,\gamma )$. As the temperature decreases, an eigenvalue of the RPA susceptibility $\chi _{\text{RPA}}$ at a specific momentum ${{\bf q}}$ turns negative, signaling an instability at this ${{\bf q}}$ vector. The associated eigenvector contains the structure of the charge instability, i.e. the CDW pattern.

According to our previous analysis, the relevant fluctuations are in the onsite and anti-symmetric bond channels and we thus study the effect of inter-site Coulomb interactions on them. With a typical NN repulsion of $V_{\text{NN}}=0.6$, the susceptibilities in various channels $\chi ^{\prime ,\prime \prime }/\Omega$ are displayed in Fig. 2b. The NN bond fluctuations are significantly enhanced at the ${{{\bf M}}}$ point, but the NN real bond susceptibility is dominant, consistent with previous studies [57,58]. The NN imaginary bond fluctuation (green dashed line) is the subdominant, while the onsite charge fluctuation is quite weak. In contrast, with a moderate NNN repulsion $V_{\text{NN}}=0.95$, the susceptibilities of imaginary bond orders are significant at the ${\bf M}$ point and the NNN imaginary bond susceptibility is much larger than the others, as shown in Fig. 2c. This indicates that the NNN repulsion can promote the imaginary bond fluctuation on the NNN bond. When both NN and NNN repulsions are substantial, the onsite charge fluctuation at ${\bf q}=0$ exceeds the bond fluctuations at the ${\bf M}$ point and becomes dominant, as shown in Fig. 2d. Meanwhile, the enhancement of the onsite charge susceptibility at the ${\bf M}$ point always remains weak, due to the aforementioned sublattice interference effect.

**Figure 3.**
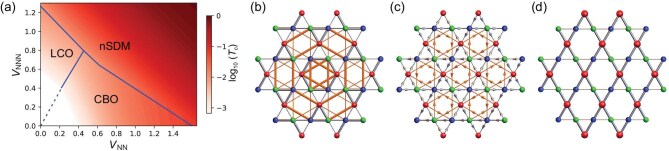
Phase diagram and real-space patterns of relevant charge orders. (a) Phase diagram of the spinless kagome lattice with inter-site Coulomb interactions at the *p*-type VH filling. Real-space patterns of three orders: (b) trihexagonal pattern of the CBO, (c) bond pattern of the LCO, (d) nematic SDM. The thick (thin) bond represents a strong (weak) hopping, the arrow represents the direction of the current and the size of sphere denotes the charge density. The transition temperature in the white region is below $10^{-3}$.

We further scrutinize the eigenvalues of $\chi _{\text{RPA}}({{\bf q}})$ to study the particle-hole instabilities with decreasing temperature. The obtained phase diagram is displayed in Fig. 3a, with color representing the transition temperatures. When the NN repulsion is dominant and the NNN repulsion is weak (region I), the susceptibility of the real bond order at three ${\bf M}$ points first diverges as the temperature decreases and the system favors the charge bond order (CBO). For a dominant NNN repulsion (region II), the imaginary bond order, i.e., loop-current order (LCO), is the leading instability. Because of the coupling between the bond order on the NN and NNN bonds, both the CBO and LCO exhibit a sizable mixture between these bonds, as shown in Fig. 3b and 3c. These particle-hole instabilities are consistent with our weak-coupling analysis, which indicates that the real bond order generates uniformly larger gaps on the Fermi surface for the NN channel and the imaginary bond order produces larger gaps on the Fermi surface for the NNN channel (details in the online supplementary material). When both NN and NNN repulsions are strong (region III), the two-fold charge order with ${{\bf q}}=0$ is favored and characterized by a mixture of onsite and symmetric bond orders (shown in Fig. 3d). The onsite order, characterized by distinct occupations on three sublattices, is dubbed as nematic sublattice density modulation (nSDM) and exhibits an electrostatic energy gain that scales linearly with the increasing inter-sublattice repulsion. When the Coulomb repulsion is relatively weak, this energy gain is small and charge bond orders predominate. However, as the repulsion strengthens, the energy benefit of the onsite charge order increases rapidly, making it the dominant configuration under conditions of strong repulsion (details in the online supplementary material).

**Figure 4.**
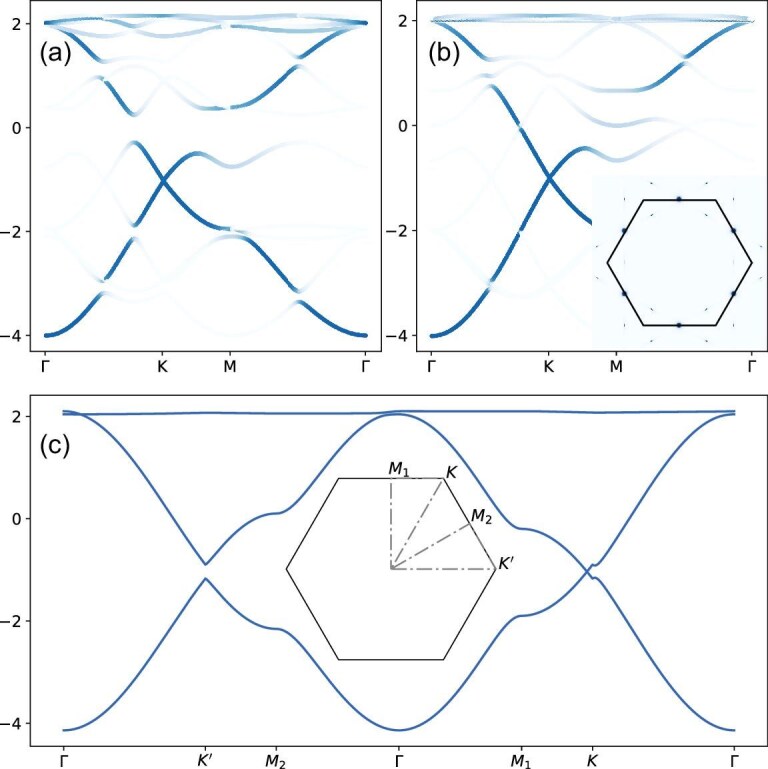
Unfolded band structures for relevant charge orders: (a) the $2\times 2$ CBO, (b) the $2\times 2$ LCO and (c) the nematic order with charge-density modulations. The NN and NNN bond order parameters in the anti-symmetric channel are $\Delta _{-,\text{NN}}=-0.12$ and $\Delta _{-,\text{NNN}}=0.07$ for the CBO and $\Delta _{-,\text{NN}}=-0.09i$ and $\Delta _{-,\text{NNN}}=0.10i$ for the LCO. For the nematic SDM phase, the adopted parameters are $\Delta _0(2,-1,-1)$ in the onsite channel with $\Delta _0=0.1$ and $\Delta _{2(3),+,\text{NN}}=0.05$ in the NN symmetrical channel. The detailed Hamiltonian is provided in the online supplementary material.

For both the CBO and LCO, the instability occurs simultaneously at three symmetry-related ${\bf M}$ points and the ground state can be determined by the analysis of Ginzburg–Landau free energy. In the CBO, the trilinear term favors the triple-${\bf M}$ phase with $2\times 2$ reconstructions and the corresponding sign of its coefficient determines the real-space pattern [51]: a negative sign favors the trihexagonal pattern and a positive sign favors the star-of-David pattern. The real-space trihexagonal configuration involving NN and NNN bonds is displayed in Fig. 3b, where the thick (thin) bond represents a strong (weak) hopping. With typical order parameters, the corresponding unfolded band structure is shown in Fig. 4a and the Fermi surface is fully gapped with a maximum gap occurring around the VHSs. For the LCO, the trilinear term vanishes due to the time-reversal symmetry, and the free energy up to quartic terms reads


(18) \begin{eqnarray*}
F_{\text{LCO}} &=& a\Psi ^2+b\Psi ^4\\
&&+\, c\left(\psi ^2_1\psi ^2_2+\psi ^2_2\psi ^2_3+\psi ^2_3\psi ^2_1\right),
\end{eqnarray*}

where $\psi _i$ is the order parameter of the LCO with the vector $\text{M}_i$ and $\Psi ^2=\sum _i\psi ^2_i$. The quadratic coefficient is $a=a_0(T-T_c)$ with $a_0>0$. The coefficient of the coupling term determines the ground state. A large positive *c* usually favors the single-${\bf M}$ phase with $1\times 2$ reconstructions, but a negative *c* favors the triple-${\bf M}$ phase with $2\times 2$ reconstructions. Figure 3c illustrates the real-space pattern of the triple-${\bf M}$  $2\times 2$ LCO with six-fold rotational symmetry, where the arrows denote the direction of the current pattern emerging in both NN and NNN bonds. Within this phase, the time-reversal symmetry is broken and the occupied band features a nontrivial Chern number and an orbital magnetism occurs. The orbital magnetic moment is correlated with the order parameter of the LCO (see Section VII within the online supplementary material for details). The unfolded band structure is displayed in Fig. 4b, and the gap opening is anisotropic: the gap along $\Gamma$-K almost vanishes, but reaches the maximum at VHSs. Distinct from the CBO, there is an additional state located at the Fermi level around VHSs. These lead to finite spectral weight at the Fermi energy along the $\Gamma$-K line and around ${\bf M}$, as observed from the Fermi surface shown in the inset of Fig. 4b. For the two-fold sublattice density modulation order, the free energy reads


(19) \begin{eqnarray*}
F_{\text{CDW}}=a^{\prime }(\rho ^2_1+\rho ^2_2)+c^{\prime }(\rho ^3_+ +\rho ^3_-),
\end{eqnarray*}

where $\rho _{1,2}$ are the two-fold order parameters and $\rho _{\pm }=\rho _1\pm i\rho _2$. Assuming that $(\rho _1,\rho _2)=\rho (\sin 2\theta , \cos 2\theta )$, the cubic term can be written as $\rho ^3\cos (6\theta )$, which is minimized by $2\theta =2n\pi /3$ for $c^{\prime }<0$ and $2\theta =(2n+1)\pi /3$ for $c^{\prime }>0$. The resulting order will break the six-fold rotational symmetry and is thus nematic. The order mainly involves charge-density modulations within the unit cell and a representative nematic real-space configuration is shown in Fig. 3d, where the large red spheres denote larger occupation and the red sublattice related bonds have stronger hopping amplitude. As shown in Fig. 4c, this nSDM order will not introduce any band fold and gap opening around the Fermi level, but will introduce anisotropic energy shifts for the VHSs.

## SUPERCONDUCTIVITY MEDIATED BY ONSITE AND BOND CHARGE DENSITY FLUCTUATIONS

**Figure 5.**
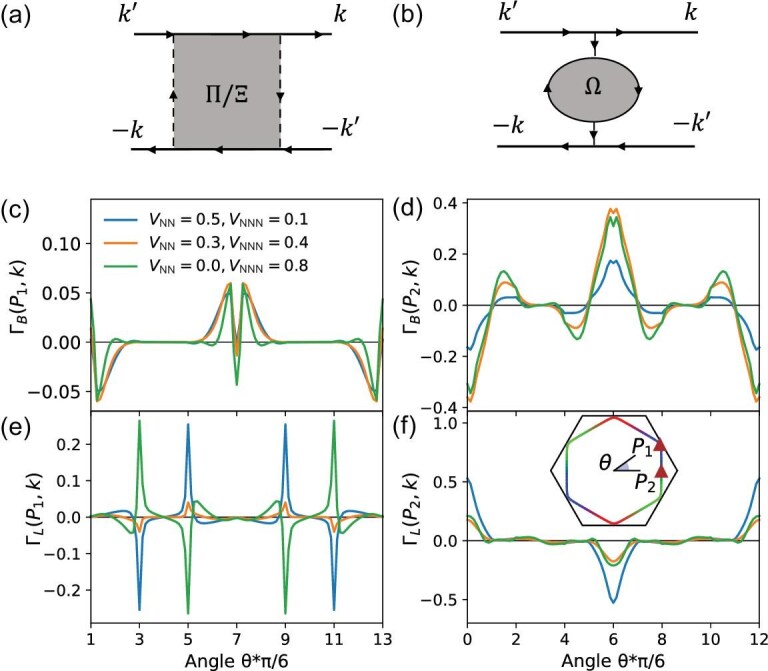
Effective Cooper pair scattering mediated by onsite and bond charge fluctuations. Feynman diagrams of the effective pairing interaction from bond (a) and onsite (b) charge fluctuations. Effective pairing interaction on the Fermi surface from bubbles (c, d) and ladders (e, f). Two reference points ${P}_1$ and ${P}_2$ are marked in the insert of (f) and $\theta$ is measured in the counterclockwise direction from the horizontal axis. The adopted chemical potential and temperature are $\mu =0.01$ and $k_B T=0.007$, respectively.

When the Fermi level moves away from VHSs, the Fermi surface nesting weakens, leading to the suppression of both onsite and bond charge orders. However, these charge fluctuations can promote particle-particle instabilities, i.e. superconductivity. In this section, we explore the induced superconducting pairing when these particle-hole orders become unstable. Based on the Feynman diagrams in Fig. 5a and b, the onsite charge fluctuation ($\Omega$) peaking at ${{\bf q}}=0$ dominantly contributes to the forward Cooper pair scattering, while the bond charge fluctuation ($\Pi / \Xi$) peaking at $\mathbf {M}$ points contributes to the Cooper pair scattering with a large momentum transfer. The effective pairing interaction vertex $\Gamma (\boldsymbol {k}, \boldsymbol {k}^{\prime })$ can be expressed as the onsite and bond charge fluctuations in the RPA approximation (details in the online supplementary material). We tune the chemical potential slightly away from VH filling and the corresponding Fermi surface is shown in the inset of Fig. 5f, where there are two representative points ${P}_1$ and ${P}_2$. We plot the effective interaction from RPA bubbles $\Gamma _{\text{B}}$ ($\Omega$) and ladders $\Gamma _{\text{L}}$ ($\Pi / \Xi$) with different inter-site Coulomb interactions in panels (c)–(f) of Fig. 5, respectively. When one momentum is fixed at point ${P}_1$, whose eigenvector is dominantly contributed by one sublattice, the effective interaction $\Gamma _{\text{B}}({P}_1,\boldsymbol {k})$ is weak and nonzero only when $\boldsymbol {k}$ is close to $\pm {P}_1$ due to the sublattice texture on the Fermi surface [66,67]. In contrast, the effective interaction $\Gamma _{\text{L}}({P}_1,\boldsymbol {k})$ is substantial and exhibits sharp peaks when $\boldsymbol {k}$ is in proximity to the other two VHSs. Moreover, the effective interactions display opposite signs in two cases where $V_{\text{NN}}$ and $V_{\text{NNN}}$ are dominant. The Cooper pair scattering between different VHSs can be exclusively mediated by the bond fluctuation $\Xi$. The NN and NNN $\Xi$ at the nesting vector feature opposite signs and thus real and imaginary bond fluctuations generate opposite effective interactions (details in the online supplementary material), featuring distinct pairing states. When one momentum is fixed at point ${P}_2$, whose eigenvector is attributed to a mixture of two sublattices, the effective interaction $\Gamma _{\text{B}}({P}_2,\boldsymbol {k})$ is large and peaks at $\boldsymbol {k}=\pm {P}_2$. Because of its anti-symmetric nature, it turns repulsive around $\boldsymbol {k}=-{P}_2$ ($\theta =\pi$), as indicated from Fig. 5d. The effective interaction $\Gamma _{\text{L}}({P}_2,\boldsymbol {k})$ mainly mediated by the bond fluctuation $\Pi$ is also significant around $\boldsymbol {k}=\pm {P}_2$ and becomes attractive around $\boldsymbol {k}=-{P}_2$, but drops to zero when the momentum transfer is large, as shown in Fig. 5f. Intriguingly, the total effective interaction $\Gamma _{\text{T}}({P}_2,\boldsymbol {k})=\Gamma _{\text{B}}({P}_2,\boldsymbol {k})+\Gamma _{\text{L}}({P}_2,\boldsymbol {k})$ for $\boldsymbol {k}$ around $-{P}_2$ is attractive with a dominant $V_{\text{NN}}$, but repulsive with a dominant $V_{\text{NNN}}$, which determines the pairing gap functions.

Near the transition temperature, the gap function can be obtained by solving the linearized gap equation


**Figure 6.**
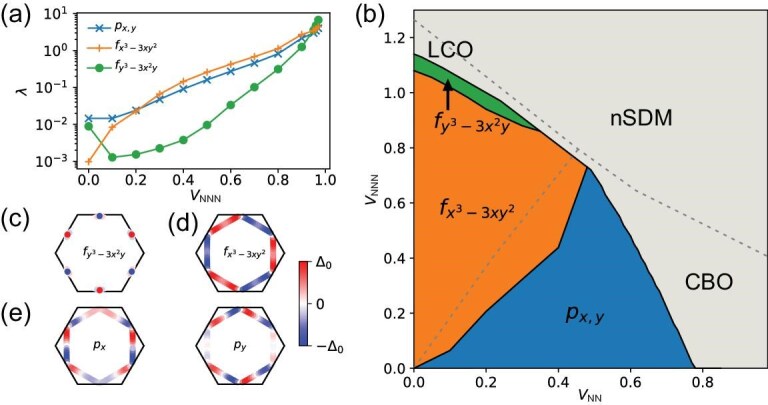
Superconducting phase diagram and leading gap functions away from the *p*-type VH filling. (a) Pairing eigenvalues $\lambda$ with a variation of $V_{\text{NNN}}$ and a fixed $V_{\text{NN}}=0.2$. (b) Phase diagram of superconductivity from the inter-site repulsions. In the upper right region, RPA charge susceptibilities diverge at the adopted temperature $k_BT=0.007$ and thus particle-hole instabilities are leading. Representative superconducting gap functions: (c) $f_{y^3-3x^2y}$ wave, (d) $f_{x^3-3xy^2}$ wave, (e) *p* wave.

(20) \begin{eqnarray*}
-\int _{\rm FS} \frac{\sqrt{3}d {\bf k^{\prime }}}{2(2 \pi )^2|v_{\text{F} }({\bf k^{\prime }})|}V^{\rm t}(\mathbf {k},\mathbf {k}^\prime ) \Delta _{i}(\mathbf {k}^{\prime })\!=\!\lambda _i\Delta _{i}(\mathbf {k}),
\end{eqnarray*}

where $v_{\text{F}}({\bf k})$ is the Fermi velocity at momentum ${\mathbf {k}}$ on the FS, and $\lambda _{i}$ denotes the pairing strength for the gap function $\Delta _{i}({\bf k})$ from the pairing interaction vertex in the triplet (t) channel, with $V^{\text{t}}(\boldsymbol {k},\boldsymbol {k}^{\prime })=\frac{1}{2}[\Gamma _{\text{T}}(\boldsymbol {k},\boldsymbol {k}^{\prime })- \Gamma _{\text{T}}(\boldsymbol {k},-\boldsymbol {k}^{\prime })]$ (for details, see the online supplementary material). Here, only triplet pairing is allowed due to the spinless model. We study the dominant pairing states based on the above equation and Fig. 6a displays the leading pairing eigenvalues with a variation of $V_{\text{NNN}}$ and a fixed $V_{\text{NN}}=0.2$. The *p*-wave state is favored for $V_{\text{NNN}}<0.2$. For an intermediate $V_{\text{NNN}}$, the $f_{x^3-3xy^2}$-wave pairing is dominant. Increasing $V_{\text{NNN}}$ further, the eigenvalue of the $f_{y^3-3yx^2}$-wave pairing increases rapidly and becomes dominant around $V_{\text{NNN}}=1.0$. The comprehensive $V_{\text{NN}}$-$V_{\text{NNN}}$ phase diagram is illustrated in Fig. 6b. Here, the two dominant *p*-wave and $f_{x^3-3xy^2}$-wave pairings lie adjacent to the CBO and LCO/nSDM, implying that their emergence is facilitated by the corresponding charge fluctuations. The corresponding gap functions are displayed in Fig. 6c–e, where the *f*-wave gaps feature a sign change with a six-fold rotation and the $p_{x,y}$-wave state is two-fold degenerate. The $p_{x,y}$-wave pairing tends to form a $p_x+ip_y$ state to maximize the superconducting condensation energy. The $f_{y^3-3yx^2}$-wave state existing in a narrow region is extremely close to the LCO, indicating that it is dominantly promoted by loop-current fluctuations. The superconducting gaps around saddle points connected by the nesting vectors have the same sign, which is dictated by the attractive pairing interaction between different VHSs. The $f_{x^3-3xy^2}$-wave pairing is generated by both the LCO and nSDM fluctuations and the sign-reversed gaps between two opposite edges are attributed to the repulsive nature of $\Gamma _{\text{T}}({P}_2,\boldsymbol {k})$ around $\boldsymbol {k}=-{P}_2$. The *p*-wave pairing is driven by the CBO fluctuations and the attractive total interaction $\Gamma _{\text{T}}({P}_2,\boldsymbol {k})$ around $\boldsymbol {k}=-{P}_2$ ensures that the gaps maintain the same sign on two opposite edges. In addition, its odd-parity nature will introduce line nodes along the $\Gamma$-K line. Nonmagnetic disorders will suppress the transition temperatures of these sign-changing pairing states, but will not change their symmetries (see the online supplementary material). In our spinless model, spin-singlet pairing is absent, and thus our results of pairing states cannot be directly applied to realistic kagome materials. However, the discussed effective Cooper scattering between VHSs can play an essential role in determining pairing symmetry within kagome materials.

## DISCUSSIONS AND CONCLUSIONS

At the *p*-type VH filling, the associated sublattice texture on the Fermi surface plays a pivotal role in determining the correlated states in the kagome lattice. The real-space $2\times 2$ modulated onsite charge order is significantly suppressed and the bond charge order gets promoted due to the sublattice interference. Owing to the unique geometry of the kagome lattice, the NN and NNN bonds are characterized by strong intrinsic real and imaginary bond fluctuations, respectively. The loop-current state can naturally emerge when there is a strong NNN repulsion. Our work demonstrates that the kagome lattice is an ideal platform to realize such loop-current states. The obtained $2\times 2$ loop-current order is in the anti-symmetric channel and breaks the translational symmetry derived from the Fermi surface nesting. It is distinct from the loop-current order in the symmetric channel with $\boldsymbol {q}=0$ at 1/3 or 2/3 fillings, where quadratic band touching is believed to be essential [27–29]. The inclusion of NNN hopping will deform the Fermi surface at VH filling, weakening Fermi surface nesting, but the associated sublattice texture remains and thus the discussed mechanism is robust. A weak NNN hopping will slightly suppress the bond order but enhance nSDM, only leading to quantitative changes in the phase diagram according to our calculations (see Section VIII within the online supplementary material).

In nonmagnetic kagome materials, there is an additional degree of freedom, i.e. the electron’s spin. In a spinful model, the onsite Coulomb repulsion becomes relevant and the onsite charge fluctuation gets enhanced as the charge density doubles. A strong NNN repulsion will further enhance the onsite CDW, rendering the LCO subleading. However, the third NN repulsion acting on the same sublattice can suppress the CDW and the LCO may still be stabilized in certain parameter space. A strong onsite Coulomb interaction can enhance the spin bond order and complicates the phase diagram, which deserves future investigation.

We discuss the potential experimental implications of the correlated states in our calculations. The obtained $2\times 2$ LCO state can be relevant in two types of kagome materials. In AV$_3$Sb$_5$, the CDW exhibits an in-plane $2\times 2$ reconstruction and TRS breaking. There are both *p*-type and *m*-type VHSs in the vicinity of the Fermi level and the multi-orbital nature and strong hybridization between V *d* orbitals and Sb *p* orbitals can enhance the inter-site repulsion in the kagome lattice [36,37]. These are consistent with the setting in our model calculations. Moreover, the multiple types of VHSs may be helpful to stabilize the LCO in the spinful case [68]. The delocalized Wannier functions on the kagome lattice due to strong *d*-*p* hybridization enhances the inter-site interaction and the multi-fold VHSs could reduce the critical interaction for the LCO. Indeed, constrained RPA calculations suggest that the NN and NNN repulsions in AV$_3$Sb$_5$ and FeGe are comparable in strength [69]. The CDW observed in AV$_3$Sb$_5$ may be attributed to the LCO and driven by inter-site Coulomb interactions. Another relevant kagome material is FeGe, which exhibits both antiferromagnetic and CDW orders. Each kagome layer is ferromagnetic and ferromagnetic splitting is large, resulting in multiple spin-polarized VHSs in proximity to the Fermi level [34,35]. This spin-polarized band is close to the adopted spinless kagome model here. The orbital magnetism associated with the LCO could account for the change in the magnetic moment upon the CDW transition in FeGe [34,35]. The nematic SDM involving onsite and symmetric bond orders in our calculations can account for the nematicity in CsTi$_3$Bi$_5$ observed by the quasi-particle interference in scanning tunneling microscope (STM) measurements [70,71]. In particular, the observed anisotropic symmetry-breaking feature in momentum space can be attributed to the nematic bond order. For other kagome materials like ScV$_6$Sn$_6$ and LaRu$_3$Si$_2$, the charge orders are not correlated with VHS-related Fermi surface nesting, and are thus out of the scape of this work [72,73]. In the three-dimensional (3D) frustrated pyrochlore lattice—a 3D counterpart of the kagome lattice formed by a network of corner-sharing tetrahedra—the revealed sublattice interference may also appear and help promote exotic charge orders, which could be relevant for 3D materials such as CeRu$_2$ and CuV$_2$S$_4$ [74–76].

In summary, our study demonstrates that the loop-current state can be stabilized within the spinless kagome lattice, driven by the pronounced imaginary bond fluctuations on NNN bonds. The uncovered sublattice texture plays a pivotal role in the formation of bond charge orders, with the accompanying sublattice interference being deeply connected to the emergence of exotic correlated states. Our findings shed light on the unique character of the kagome lattice and propose a new mechanism for realizing exotic orders in kagome-based materials, which can be applied to other lattices with multiple sublattices.

## Supplementary Material

nwaf414_Supplemental_File

## Data Availability

The data that support the findings of this study are available from the corresponding authors upon request.
