# Supplementary material for: Exotic charge-density waves and superconductivity on the kagome lattice
Source: Natl Sci Rev. 2025 Sep 29;12(11):nwaf414. doi: 10.1093/nsr/nwaf414 (PMC12629243; doi:10.1093/nsr/nwaf414)
Supplement: nwaf414_Supplemental_File [file nwaf414_supplemental_file.pdf]

Supplemental Material for "Exotic charge density waves and superconductivity on the Kagome Lattice"

Rui-Qing Fu,[1,2] Jun Zhan,[3,2] Hendrik Hohmann,[4] Matteo Dürrnagel,[4] Ronny Thomale,[4] Jiangping Hu,[3] Ziqiang Wang,[5,*] Sen Zhou,[1,2,6,†] and Xianxin Wu[1,‡]

[1]*CAS Key Laboratory of Theoretical Physics, Institute of Theoretical Physics, Chinese Academy of Sciences, Beijing 100190, China*
[2]*School of Physical Sciences, University of Chinese Academy of Sciences, Beijing 100049, China*
[3]*Institute of Physics, Chinese Academy of Sciences, Beijing 100190, China*
[4]*Institut für Theoretische Physik und Astrophysik, Universität Würzburg, Am Hubland Campus Süd, Würzburg 97074, Germany*
[5]*Department of Physics, Boston College, Chestnut Hill, Massachusetts 02467, USA*
[6]*CAS Center for Excellence in Topological Quantum Computation, University of Chinese Academy of Sciences, Beijing 100049, China*

In this Supplementary Material, we discuss the details about calculations of the onsite and bond charge susceptibility, RPA formalism for analysis of charge instabilities and charge fluctuation mediated pairing interaction. The lattice convention in this SM is the same with the main text.

I. ONSITE AND BOND CHARGE OPERATORS AND SUSCEPTIBILITY

A. Onsite and bond charge operators

We explore the competing charge order in the kagome lattice and the relevant orders are in the onsite and bond channels. The onsite charge operator is

$$n_\alpha(\boldsymbol{r}) = c^\dagger_{\alpha,\boldsymbol{r}} c_{\alpha,\boldsymbol{r}}, \tag{S1}$$

with α being the sublattice index. After the Fourier transformation, the operation in the momentum space reads,

$$n_\alpha(\boldsymbol{q}) = \frac{1}{\sqrt{N}} \sum_{\boldsymbol{k}} c^\dagger_{\alpha,\boldsymbol{k}+\boldsymbol{q}} c_{\alpha,\boldsymbol{q}}. \tag{S2}$$

We additionally consider bond charge modulation, i.e. charge bond order, on NN and NNN bonds. Due to the unique geometry of the kagome lattice, within each unit cell there are two NN (NNN) bonds along the direction parallel (perpendicular) to each basis vector $\boldsymbol{a}_\alpha$, and they all connect two distinct sublattices β and γ, with the Levi-civta symbol satisfying $\epsilon_{\alpha\beta\gamma} = 1$, i.e., $(\alpha, \beta, \gamma) = (1, 2, 3)$, (2, 3, 1), and (3, 1, 2). The bond operator involving β, γ sublattices and its complex conjugate are defined as,

$$\begin{aligned} B_{\alpha,s,\eta}(\boldsymbol{r_\beta}) &= \frac{1}{2}(c^\dagger_{\beta,r_\beta} c_{\gamma,r_\beta+l_{\alpha,\eta}} + s c^\dagger_{\beta,r_\beta} c_{\gamma,r_\beta-l_{\alpha,\eta}}), \\ B^\dagger_{\alpha,s,\eta}(\boldsymbol{r_\beta}) &= \frac{1}{2}(c^\dagger_{\gamma,r_\beta+l_{\alpha,\eta}} c_{\beta,r_\beta} + s c^\dagger_{\gamma,r_\beta l_{\alpha,\eta}} c_{\beta,r_\beta}), \end{aligned} \tag{S3}$$

with $s = +/-$ denoting the symmetric/antisymmetric channel and the connecting vectors being $l_{\alpha,1} = \frac{1}{2}\boldsymbol{a}_\alpha$, $l_{\alpha,2} = \frac{1}{2}(\boldsymbol{a}_\beta - \boldsymbol{a}_\gamma)$. The Fourier transformation of $B_{\alpha,s,\eta}(\boldsymbol{r})$ and $B^\dagger_{\alpha,s,\eta}(\boldsymbol{r})$ are given by

$$\begin{aligned} \tilde{B}_{\alpha,s,\eta}(\boldsymbol{q}) &= \frac{1}{\sqrt{N}} \sum_{\boldsymbol{k}} \tilde{f}_{\alpha,s,\eta}(\boldsymbol{k}) c^\dagger_{\beta,\boldsymbol{k}+\boldsymbol{q}} c_{\gamma,\boldsymbol{k}}, \\ \tilde{B}^\dagger_{\alpha,s,\eta}(\boldsymbol{q}) &= \frac{1}{\sqrt{N}} \sum_{\boldsymbol{k}} \tilde{f}^*_{\alpha,s,\eta}(\boldsymbol{k}+\boldsymbol{q}) c^\dagger_{\gamma,\boldsymbol{k}+\boldsymbol{q}} c_{\beta,\boldsymbol{k}}. \end{aligned} \tag{S4}$$

* wangzi@bc.edu
† zhousen@itp.ac.cn
‡ xxwu@itp.ac.cn

Here, the complex form factors are given by $\tilde{f}_{\alpha,+,\eta}(\boldsymbol{k}) = \cos(\boldsymbol{k}\cdot\boldsymbol{l_{\alpha,\eta}})$ and $\tilde{f}_{\alpha,-,\eta}(\boldsymbol{k}) = -i\sin(\boldsymbol{k}\cdot\boldsymbol{l_{\alpha,\eta}})$. The tilde symbol on $\tilde{B}_{\alpha,s,\eta}$ and $\tilde{f}_{\alpha,s,\eta}$ represents the complex form factor obtained directly from the Fourier transform. For the convenience of susceptibility calculations in the following, we further introduce the real form factors $f_{\alpha,+,\eta}(\boldsymbol{k}) = \cos(\boldsymbol{k}\cdot\boldsymbol{l_{\alpha,\eta}})$ and $f_{\alpha,-,\eta}(\boldsymbol{k}) = \sin(\boldsymbol{k}\cdot\boldsymbol{l_{\alpha,\eta}})$ without the tilde symbol. The bond operators defined by these real form factors are

$$\begin{aligned} B_{\alpha,s,\eta}(\boldsymbol{q}) &= \frac{1}{\sqrt{N}}\sum_{\boldsymbol{k}} f_{\alpha,s,\eta}(\boldsymbol{k}) c^{\dagger}_{\beta,\boldsymbol{k}+\boldsymbol{q}} c_{\gamma,\boldsymbol{k}}, \\ B^{\dagger}_{\alpha,s,\eta}(\boldsymbol{q}) &= \frac{1}{\sqrt{N}}\sum_{\boldsymbol{k}} f_{\alpha,s,\eta}(\boldsymbol{k}+\boldsymbol{q}) c^{\dagger}_{\gamma,\boldsymbol{k}+\boldsymbol{q}} c_{\beta,\boldsymbol{k}}. \end{aligned} \tag{S5}$$

The relation between bond operators with and without tilde symbol is given by,

$$\begin{aligned} \tilde{B}_{\alpha,+,\eta}(\boldsymbol{q}) &= B_{\alpha,+,\eta}(\boldsymbol{q}), \\ \tilde{B}^{\dagger}_{\alpha,+,\eta}(\boldsymbol{q}) &= B^{\dagger}_{\alpha,+,\eta}(\boldsymbol{q}), \\ \tilde{B}_{\alpha,-,\eta}(\boldsymbol{q}) &= -iB_{\alpha,-,\eta}(\boldsymbol{q}), \\ \tilde{B}^{\dagger}_{\alpha,-,\eta}(\boldsymbol{q}) &= iB^{\dagger}_{\alpha,-,\eta}(\boldsymbol{q}). \end{aligned} \tag{S6}$$

Note that only $\tilde{B}$ operators are physical and the sign difference in the anti-symmetric channel should be noticed. From the above definitions, it can be easily shown that

$$\begin{aligned} [n(\boldsymbol{q})]^{\dagger} &= n(-\boldsymbol{q}), \\ [B_{\alpha,s,\eta}(\boldsymbol{q})]^{\dagger} &= B^{\dagger}_{\alpha,s,\eta}(-\boldsymbol{q}). \end{aligned} \tag{S7}$$

B. Bare Susceptibilities for the relevant charge orders

To investigate the intrinsic fluctuations of different charge orders and their coupling, we calculate the corresponding susceptibilities defined as,

$$\chi_{pq}(\mathbf{q}, i\omega_n) = \int_0^{\beta} d\tau e^{i\omega_n\tau}\langle T_\tau O_o(\boldsymbol{q},\tau)[O_{o'}(\boldsymbol{q},0)]^{\dagger}\rangle = \int_0^{\beta} d\tau e^{i\omega_n\tau}\langle T_\tau O_o(\mathbf{q},\tau) O^{\dagger}_{o'}(-\boldsymbol{q},0)\rangle. \tag{S8}$$

Here the operator O_o runs over the 27 charge orders mentioned above, i.e. $O_o = [(B_{1,+,\mathrm{NN}}, B^{\dagger}_{1,+,\mathrm{NN}}, B_{1,-,\mathrm{NN}}, B^{\dagger}_{1,-,\mathrm{NN}}), (1 \to 2,3), (\mathrm{NN} \to \mathrm{NNN}), n_1, n_2, n_3]$. The analytical expressions of the bare susceptibilities in all channels are given by,

$$\left\langle n_\alpha [n_{\alpha'}]^{\dagger}\right\rangle(\boldsymbol{q}, i\omega_n) = -\frac{1}{N}\sum_{k\mu\nu} a^{\alpha*}_{\mu}(\boldsymbol{k}+\boldsymbol{q}) a^{\alpha}_{\nu}(\boldsymbol{k}) a^{\alpha'*}_{\nu}(\boldsymbol{k}) a^{\alpha'}_{\mu}(\boldsymbol{k}+\boldsymbol{q}) \frac{n_F(E_\mu(\boldsymbol{k}+\boldsymbol{q})) - n_F(E_\nu(\boldsymbol{k}))}{i\omega_n + E_\mu(\boldsymbol{k}+\boldsymbol{q}) - E_\nu(\boldsymbol{k})}, \tag{S9}$$

$$\left\langle n_\alpha [B_{2m'-1}]^{\dagger}\right\rangle(\boldsymbol{q}, i\omega_n) = -\frac{1}{N}\sum_{k\mu\nu} a^{\alpha*}_{\mu}(\boldsymbol{k}+\boldsymbol{q}) a^{\alpha}_{\nu}(\boldsymbol{k}) a^{\gamma'*}_{\nu}(\boldsymbol{k}) a^{\beta'}_{\mu}(\boldsymbol{k}+\boldsymbol{q}) f_q(\boldsymbol{k}) \frac{n_F(E_\mu(\boldsymbol{k}+\boldsymbol{q})) - n_F(E_\nu(\boldsymbol{k}))}{i\omega_n + E_\mu(\boldsymbol{k}+\boldsymbol{q}) - E_\nu(\boldsymbol{k})}, \tag{S10}$$

$$\left\langle n_\alpha [B_{2m'}]^{\dagger}\right\rangle(\boldsymbol{q}, i\omega_n) = -\frac{1}{N}\sum_{k\mu\nu} a^{\alpha*}_{\mu}(\boldsymbol{k}+\boldsymbol{q}) a^{\alpha}_{\nu}(\boldsymbol{k}) a^{\beta'*}_{\nu}(\boldsymbol{k}) a^{\gamma'}_{\mu}(\boldsymbol{k}+\boldsymbol{q}) f_q(\boldsymbol{k}+\boldsymbol{q}) \frac{n_F(E_\mu(\boldsymbol{k}+\boldsymbol{q})) - n_F(E_\nu(\boldsymbol{k}))}{i\omega_n + E_\mu(\boldsymbol{k}+\boldsymbol{q}) - E_\nu(\boldsymbol{k})}, \tag{S11}$$

$$\left\langle B_{2m-1} [B_{2m'-1}]^{\dagger}\right\rangle(\boldsymbol{q}, i\omega_n) = -\frac{1}{N}\sum_{k\mu\nu} f_p(\boldsymbol{k}) a^{\beta*}_{\mu}(\boldsymbol{k}+\boldsymbol{q}) a^{\gamma}_{\nu}(\boldsymbol{k}) a^{\gamma'*}_{\nu}(\boldsymbol{k}) a^{\beta'}_{\mu}(\boldsymbol{k}+\boldsymbol{q}) f_q(\boldsymbol{k}) \frac{n_F(E_\mu(\boldsymbol{k}+\boldsymbol{q})) - n_F(E_\nu(\boldsymbol{k}))}{i\omega_n + E_\mu(\boldsymbol{k}+\boldsymbol{q}) - E_\nu(\boldsymbol{k})}, \tag{S12}$$

$$\left\langle B_{2m-1} [B_{2m'}]^{\dagger}\right\rangle(\boldsymbol{q}, i\omega_n) = -\frac{1}{N}\sum_{k\mu\nu} f_p(\boldsymbol{k}) a^{\beta*}_{\mu}(\boldsymbol{k}+\boldsymbol{q}) a^{\gamma}_{\nu}(\boldsymbol{k}) a^{\beta'*}_{\nu}(\boldsymbol{k}) a^{\gamma'}_{\mu}(\boldsymbol{k}+\boldsymbol{q}) f_q(\boldsymbol{k}+\boldsymbol{q}) \frac{n_F(E_\mu(\boldsymbol{k}+\boldsymbol{q})) - n_F(E_\nu(\boldsymbol{k}))}{i\omega_n + E_\mu(\boldsymbol{k}+\boldsymbol{q}) - E_\nu(\boldsymbol{k})}, \tag{S13}$$

$$\left\langle B_{2m} [B_{2m'-1}]^{\dagger}\right\rangle(\boldsymbol{q}, i\omega_n) = -\frac{1}{N}\sum_{k\mu\nu} f_p(\boldsymbol{k}+\boldsymbol{q}) a^{\gamma*}_{\mu}(\boldsymbol{k}+\boldsymbol{q}) a^{\beta}_{\nu}(\boldsymbol{k}) a^{\gamma'*}_{\nu}(\boldsymbol{k}) a^{\beta'}_{\mu}(\boldsymbol{k}+\boldsymbol{q}) f_q(\boldsymbol{k}) \frac{n_F(E_\mu(\boldsymbol{k}+\boldsymbol{q})) - n_F(E_\nu(\boldsymbol{k}))}{i\omega_n + E_\mu(\boldsymbol{k}+\boldsymbol{q}) - E_\nu(\boldsymbol{k})}, \tag{S14}$$

$$\left\langle B_{2m} [B_{2m'}]^{\dagger}\right\rangle(\boldsymbol{q}, i\omega_n) = -\frac{1}{N}\sum_{k\mu\nu} f_p(\boldsymbol{k}+\boldsymbol{q}) a^{\gamma*}_{\mu}(\boldsymbol{k}+\boldsymbol{q}) a^{\beta}_{\nu}(\boldsymbol{k}) a^{\beta'*}_{\nu}(\boldsymbol{k}) a^{\gamma'}_{\mu}(\boldsymbol{k}+\boldsymbol{q}) f_q(\boldsymbol{k}+\boldsymbol{q}) \frac{n_F(E_\mu(\boldsymbol{k}+\boldsymbol{q})) - n_F(E_\nu(\boldsymbol{k}))}{i\omega_n + E_\mu(\boldsymbol{k}+\boldsymbol{q}) - E_\nu(\boldsymbol{k})}, \tag{S15}$$

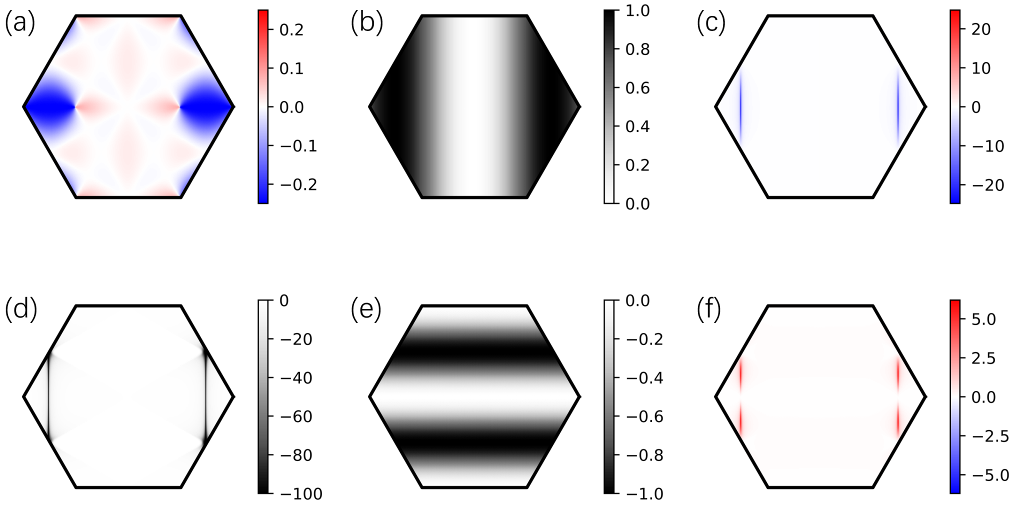

Figure S1. Detailed analysis of the sign of Ξ-type susceptibility on the NN and NNN bond: (a) $a_2^{\gamma}(\boldsymbol{k})a_2^{\beta}(\boldsymbol{k}+\mathbf{M}_1)a_2^{\gamma}((\boldsymbol{k}+\mathbf{M}_1)a_2^{\beta}(\boldsymbol{k})$. (d) $\frac{n_F(E_2(\boldsymbol{k}))-n_F(E_2((\boldsymbol{k}+\mathbf{M}_1))}{E_2(\boldsymbol{k})-E_2((\boldsymbol{k}+\mathbf{M}_1))}$. (b) NN form factor $\sin(\boldsymbol{k}\cdot\boldsymbol{l}_{\alpha,nn})\sin((\boldsymbol{k}+\mathbf{M}_1)\cdot l_{\alpha,nn})$ (e) NNN form factor $\sin(\boldsymbol{k}\cdot\boldsymbol{l}_{\alpha,nnn})\sin((\boldsymbol{k}+\mathbf{M_1})\cdot\boldsymbol{l}_{\alpha,nnn})$. (c) Contribution to $\Pi_{2;nn,nn}$ from each k-point in 1st BZ. (f) Contribution to $\Pi_{2;nnn,nnn}$

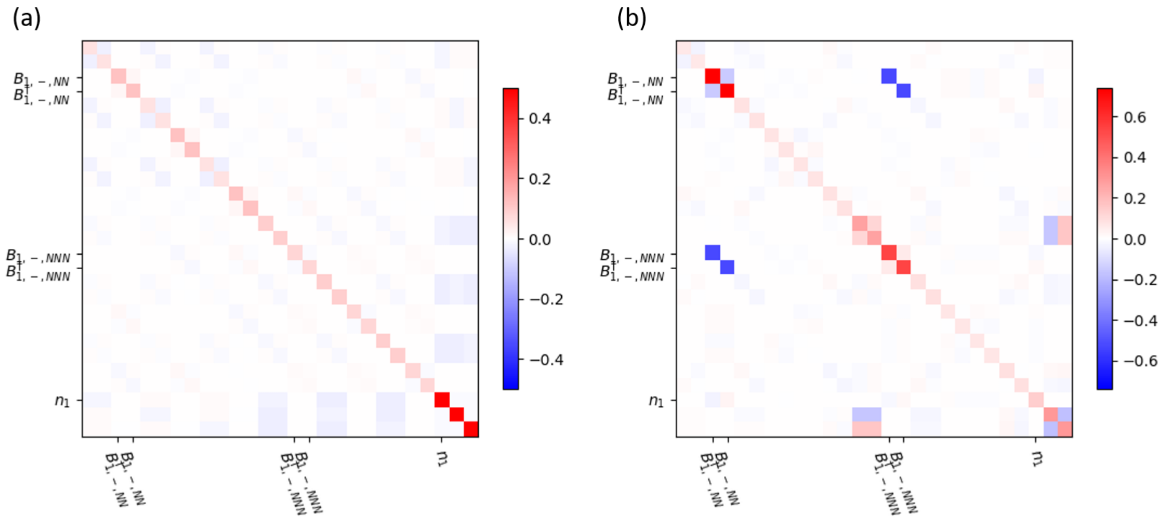

Figure S2. Bare 27 × 27 susceptibility matrix $\chi(\boldsymbol{q})$ at $\boldsymbol{\Gamma}$ point(a) and $\boldsymbol{M_1}$ point(b) with the operator order O_o = $[(B_{1,+,\mathrm{NN}}, B^{\dagger}_{1,+,\mathrm{NN}}, B_{1,-,\mathrm{NN}}, B^{\dagger}_{1,-,\mathrm{NN}}), (1 \to 2, 3), (\mathrm{NN} \to \mathrm{NNN}), n_1, n_2, n_3]$.

where $\langle O_o[O_{o'}]^\dagger\rangle$ denotes the corresponding bare susceptibility. Here μ/ν is the band index and $a^{\alpha}_{\mu}(\boldsymbol{k})$ is the α-th element of the μ-th eigenvector with corresponding index obtained from tight-binding Hamiltonian. $B_{o/o'}$ is the bond operator in o/o'-th row(column) basis, which corresponds to $(\beta,\gamma,p)/(\beta',\gamma',q)$ indices on the right site of these equations. Under this convention, B_{2m} is of the $B^\dagger$ type and B_{2m-1} is of the B type bond operator. The susceptibility of onsite charge orders belongs to the Ω-type term in main text. The operators of charge bond order (CBO) and loop current order (LCO) phase are the combination of above bond operators. The real (B') and imaginary (B'') bond operators can be expressed using defined operators,

$$B'_{\alpha,+,\eta}(\boldsymbol{q}) = \frac{1}{2}(\tilde{B}^\dagger_{\alpha,+,\eta}(\boldsymbol{q}) + \tilde{B}_{\alpha,+,\eta}(\boldsymbol{q})) = \frac{1}{2}(B^\dagger_{\alpha,+,\eta}(\boldsymbol{q}) + B_{\alpha,+,\eta})(\boldsymbol{q}), \tag{S16}$$

$$B''_{\alpha,+,\eta}(\boldsymbol{q}) = \frac{1}{2}(\tilde{B}_{\alpha,+,\eta}(\boldsymbol{q}) - \tilde{B}^\dagger_{\alpha,+,\eta}(\boldsymbol{q})) = \frac{1}{2}(B_{\alpha,+,\eta}(\boldsymbol{q}) - B^\dagger_{\alpha,+,\eta}(\boldsymbol{q})), \tag{S17}$$

$$B'_{\alpha,-,\eta}(\boldsymbol{q}) = \frac{1}{2}(\tilde{B}^\dagger_{\alpha,-,\eta}(\boldsymbol{q}) + \tilde{B}_{\alpha,-,\eta}(\boldsymbol{q})) = -\frac{i}{2}(B_{\alpha,-,\eta}(\boldsymbol{q}) - B^\dagger_{\alpha,-,\eta}(\boldsymbol{q})), \tag{S18}$$

$$B''_{\alpha,-,\eta}(\boldsymbol{q}) = \frac{1}{2}(\tilde{B}_{\alpha,-,\eta}(\boldsymbol{q}) - \tilde{B}^\dagger_{\alpha,-,\eta}(\boldsymbol{q})) = \frac{i}{2}(B_{\alpha,-,\eta}(\boldsymbol{q}) + B^\dagger_{\alpha,-,\eta}(\boldsymbol{q})). \tag{S19}$$

The sign in anti-symmetric order is different from symmetric one because we dropped the $-i$ coefficient in antisymmetric bond operators. Therefore, their static susceptibilities can be expressed using the above χ_{pq} terms,

$$\chi'_{mm'}(\boldsymbol{q}) = \frac{1}{4}(\langle B_{2m-1}[B_{2m'-1}]^\dagger\rangle + \langle B_{2m}[B_{2m'}]^\dagger\rangle + \langle B_{2m-1}[B_{2m'}]^\dagger\rangle + \langle B_{2m}[B_{2m'-1}]^\dagger\rangle)(\boldsymbol{q}), \tag{S20}$$

$$\chi''_{mm'}(\boldsymbol{q}) = \frac{1}{4}(\langle B_{2m-1}[B_{2m'-1}]^\dagger\rangle + \langle B_{2m}[B_{2m'}]^\dagger\rangle - \langle B_{2m-1}[B_{2m'}]^\dagger\rangle - \langle B_{2m}[B_{2m'-1}]^\dagger\rangle)(\boldsymbol{q}), \tag{S21}$$

when $m, m' \in$ odd represent symmetric bond operators and

$$\chi'_{mm'}(\boldsymbol{q}) = \frac{1}{4}(\langle B_{2m-1}[B_{2m'-1}]^\dagger\rangle + \langle B_{2m}[B_{2m'}]^\dagger\rangle - \langle B_{2m-1}[B_{2m'}]^\dagger\rangle - \langle B_{2m}[B_{2m'-1}]^\dagger\rangle)(\boldsymbol{q}), \tag{S22}$$

$$\chi''_{mm'}(\boldsymbol{q}) = \frac{1}{4}(\langle B_{2m-1}[B_{2m'-1}]^\dagger\rangle + \langle B_{2m}[B_{2m'}]^\dagger\rangle + \langle B_{2m-1}[B_{2m'}]^\dagger\rangle + \langle B_{2m}[B_{2m'-1}]^\dagger\rangle)(\boldsymbol{q}), \tag{S23}$$

when $m, m' \in$ even represent anti-symmetric bond operators. The first two terms on the right hand side of the equation belong to the Π-type terms in main text, while the latter two terms are Ξ-type terms. For the susceptibility of a specific bond order, $m = m'$, two Π are equal, as are the two Ξ terms. It is evident that the relative strength of susceptibilities for real and imaginary bond orders are fully determined by the sign of the Ξ.

Due to the perfect Fermi surface nesting at the p-type VH filling, we examine the characteristics of bare susceptibilities at two pertinent vectors $\boldsymbol{q} = 0, \mathbf{M}$ based on the above expressions. For $\boldsymbol{q} = 0$, the six edges of the Fermi surface in Fig.1(b) mainly contribute to the susceptibilities (as indicated by matrix elements in Eq.S9) and $\Omega^0_{\alpha\alpha}$ is dominant due to the pure sublattice nature at three VHSs with diverging density of states (DOS). In contrast, each vertex of the bond susceptibility bubble involves two different sublattices (as indicated by matrix elements in Eq.S12,S13,S14,S15) and the contribution from VHSs vanishes due to the sublattice-resolved eigenvectors in Green functions, leading to minimal values for $\Pi^0_{mn}(0)$. For $\boldsymbol{q} = \mathbf{M}$, however, the behaviors are the opposite. Taking $\boldsymbol{q} = \boldsymbol{Q}_1$ as an example, the susceptibilities are predominantly contributed by two edges connected by $\boldsymbol{Q}_1$ shown in Fig.1(b), as dictated by the Lindhard function (see Eq.S12,S13,S14,S15). As the $\boldsymbol{Q}_1$ always connects two VHSs with distinct sublattices, the contribution of these VHSs in $\Omega^0_{\alpha\alpha'}$ vanishes and only other segments away from VHSs with small DOS can contribute, resulting a small $\Omega^0_{\alpha\alpha'}(\boldsymbol{Q}_1)$. On the contrary, VHSs can contribute to Π^0_{mm}, leading to a significant $\Pi^0_{mm}(\boldsymbol{Q}_1)$. As the Green functions in the Ξ^0_{mm} involves mixed sublattices in both electron lines, the magnitude of $\Xi^0_{mm}(\boldsymbol{Q}_1)$ is small. Consequently, this unique sublattice texture at the p-type VH filling suppresses the onsite charge fluctuations with $\boldsymbol{q} = \mathbf{M}$ but promote pronounced bond charge fluctuations. This behavior in the kagome lattice markedly differs from that observed in the triangular and honeycomb lattices, where onsite charge fluctuations are dominant. Moreover, for $\boldsymbol{k}$ points on the two edges connected by the nesting vectors, $\boldsymbol{k}\cdot\boldsymbol{l}_{\alpha,\mathrm{NN}} = \pm\pi/2$ and thus the NN symmetric form factor vanishes and NN anti-symmetric one reaches the maximum. The NNN symmetric form factor also vanishes at the VHSs due to $\boldsymbol{k}_{\beta,\gamma}\cdot\boldsymbol{l}_{\alpha,\mathrm{NNN}} = \pm\pi/2$, weakening the corresponding bond fluctuations. Consequently, the anti-symmetric bond fluctuations on both NN and NNN bonds at the $\mathbf{M}$ point dominate over the corresponding symmetric conterparts.

We further analyze the Ξ susceptibility in details. From the definition of bare susceptibilities we find that the only difference between NN and NNN bond order is the form factor. The susceptibility is mainly contributed by the band close to the Fermi level. To reveal the difference between susceptibilites Ξ on NN and NNN bonds, we plot the product of matrix elements, Lindhard function and form factors of the susceptibility in the 2D Brillouinz zone, respectively. From the product of matrix elments and the Lindhard

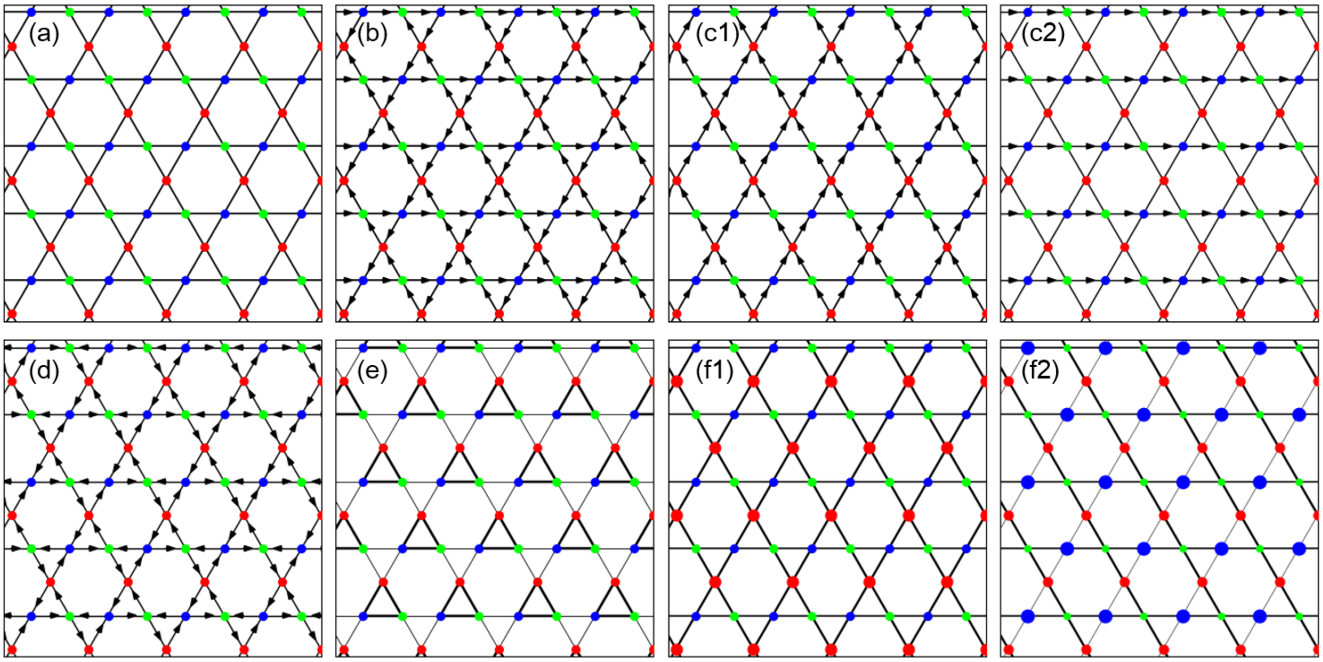

Figure S3. Possible charge orders at $\boldsymbol{q} = \mathbf{0}$ for each irreducible representation of C_{6v} group (a) A_1 (b) B_1 (c) E_1 (d) A_2 (e) B_2 (f) E_2. The radius of red, blue and green circles are onsite charge density of $1, 2, 3$ sublattices. The thickness of black bonds show the hopping amplitude. Arrows represent spontaneous current inter sublattices.

function, it is evident that the $\Xi^0_{1,-,\eta}(\mathbf{M}_1)$ susceptibility are mainly contributed by $\boldsymbol{k}$ points near the two opposite Fermi surface segments connected by the nesting vector $\boldsymbol{Q}_1$. Interestingly, the form factors for the NN and NNN bonds on these edges have the opposite sign are different so that NN and NNN, as shown in Fig.S1 (c),(d). This difference results in opposite $\Xi^0_{1,-,\eta}(\mathbf{M}_1)$ values for the NN and NNN bonds, determined by the unique kagome geometry. Note for $\boldsymbol{q} = \mathbf{M}_1$ and $\beta/\gamma = 2/3$ or $3/2$, the product of matrix element $a_2^{\gamma *}(\boldsymbol{k}+\mathbf{M}_1)a_2^{\beta}(\boldsymbol{k})a_2^{\gamma *}(\boldsymbol{k})a_2^{\beta}(\boldsymbol{k}+\mathbf{M}_1) = 0$ at two VHSs $\boldsymbol{k} = \mathbf{M}_2, \mathbf{M}_3$ and thus VHS makes no contribution to the Ξ susceptibility, as mentioned before. Therefore, the patch model involving only VHSs can fail to capture the difference between flucuations between real and imaginary bond orders.

After above analysis, we numerically illustrate the full bare susceptibility matrix elements at $\mathbf{M}_1$ and Γ point in Fig.S2 (a) and (b), respectively. At the Γ point the susceptibility matrix is dominated by its onsite component (Ω type) while bond susceptibilities are much weaker. The onsite order also exhibit some coupling with symmetric bond orders. At the $\boldsymbol{M_1}$ point, the susceptibilities of symmetric bond orders are vanishing small and those of anti-symmetric bond orders are dominant (larger than the onsite susceptibility Ω). It is apparent the susceptibilities Ξ on the NN and NNN bonds have the opposite signs, consistent with our above analysis. In addition, the coupling between NN and NNN bonds are also strong, indicating a strong mixing between them.

II. CLASSIFICATION OF CDW ORDER WITH Q = 0, M IN KAGOME LATTICE

In this section, we discussion the classification of relevant charge order in kagome lattice within our calculations. For $\boldsymbol{Q} = \Gamma/M$ point, the little group is C_{6v}/C_{2v} and the corresponding character tables are shown in TableII. For $\boldsymbol{Q} = 0$, there are five nontrivial irrep. except A_1 and the corresponding pattern of charge orders are displayed in Fig.S3. Two of them (e,f) belong to the real bond orders and three of them (b,c,d) belong to the imaginary bond orders. While, for $\boldsymbol{Q} = \mathbf{M}$, there are four kinds of pattern of charge orders, shown in Fig.S4(a),(b),(d),(e). In the symmetric and antisymmetric channels, there are both real and imaginary bond orders. Further combining order at three $\mathbf{M}$ points, we will have $3\boldsymbol{Q}$ order and the corresponding pattern are displayed in Fig.S4 (c) and (f).

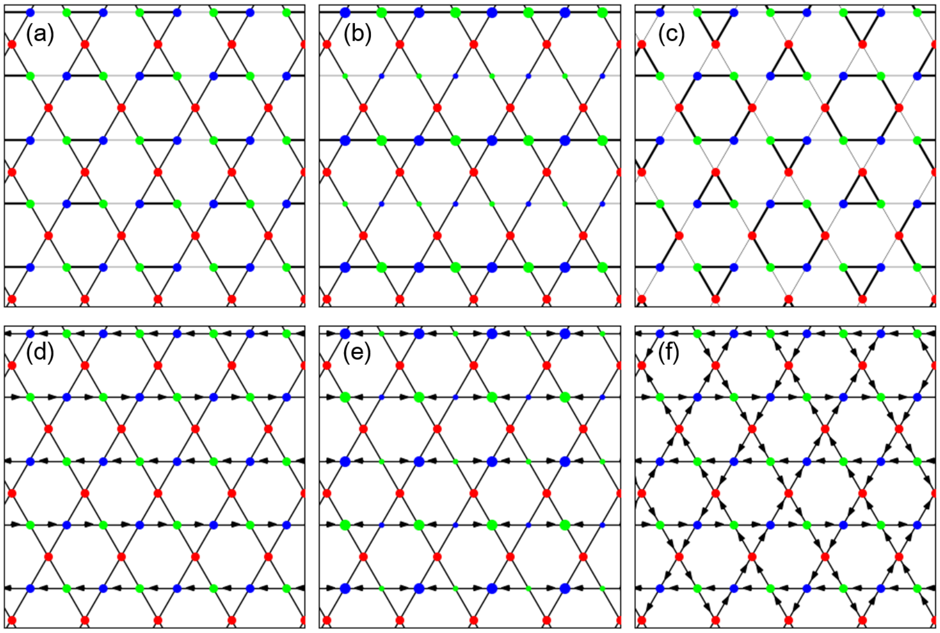

Figure S4. Possible charge orders at $\boldsymbol{q} = \boldsymbol{M}$ for each irreducible representation of C_{2v} group (a) A_1 (b) B_1 (d) A_2 (e) B_2. (c) and (f) are $3\boldsymbol{Q}$ orders of (a) and (d), respectively.

	E	$2C_6$	$2C_3$	C_2	$3\sigma_v$	$3\sigma_d$
A_1	1	1	1	1	1	1
A_2	1	1	1	1	-1	-1
B_1	1	-1	1	-1	1	-1
B_2	1	-1	1	-1	-1	1
E_1	2	1	-1	-2	0	0
E_2	2	-1	-1	2	0	0

	E	C_2	σ_v	σ_d
A_1	1	1	1	1
A_2	1	1	-1	1
B_1	1	-1	1	-1
B_2	1	-1	-1	1

Table I. Character tables of C_{6v} at $\boldsymbol{Q} = 0$ and C_{2v} at $\boldsymbol{Q} = \boldsymbol{M}$ point groups.

III. RPA FORMALISM FOR THE ONSITE AND BOND CHARGE SUSCEPBILITIES

In order to analyze the charge instability beyond the mean field, we adopted the RPA formalism. At the RPA level, the charge susceptibility is usually obtained by considering the geometric summation of bubble-type type diagrams, where onsite and bond orders usually decoupled. To treat them on equal footing, we consider both bubble and ladder diagrams with interactions. However, as interactions are nonlocal and thus the internal interaction lines involve internal momentum, the analytic summation of such ladder diagrams are difficult. Here, the special properties of inter-site repulsion disscussed in the following allows to achieve it in a matrix form for our case [S1].

To deal with the interaction lines carrying internal momentum in ladder diagrams, we decouple their form factors into bond form factors using trigonometric identities,

$$\cos((\boldsymbol{k}-\boldsymbol{k}')\cdot\boldsymbol{l}) = \cos(\boldsymbol{k}\cdot\boldsymbol{l})\cos(\boldsymbol{k}'\cdot\boldsymbol{l}) + \sin(\boldsymbol{k}\cdot\boldsymbol{l})\sin(\boldsymbol{k}'\cdot\boldsymbol{l}), , \tag{S24}$$

$$\cos((\boldsymbol{k}-\boldsymbol{k}')\cdot\boldsymbol{l}) = \cos((\boldsymbol{k}+\boldsymbol{q})\cdot\boldsymbol{l})\cos((\boldsymbol{k}'+\boldsymbol{q})\cdot\boldsymbol{l}) + \sin((\boldsymbol{k}+\boldsymbol{q})\cdot\boldsymbol{l})\sin((\boldsymbol{k}'+\boldsymbol{q})\cdot\boldsymbol{l}). \tag{S25}$$

With this decoupling, two internal momenta are separated and two additional vertices appear, as schematically demonstrated in

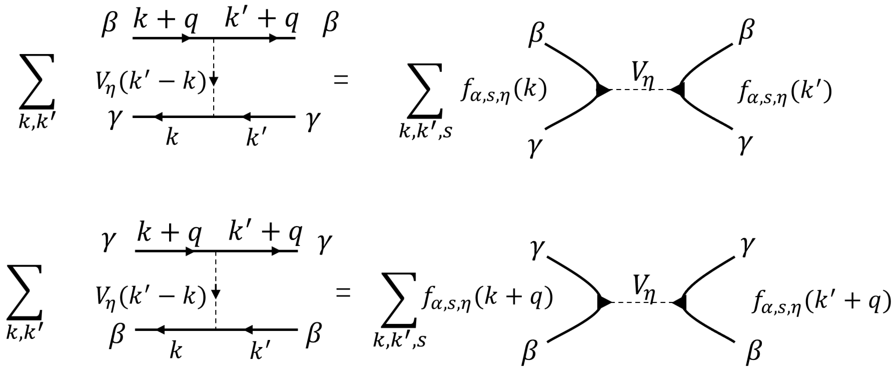

Figure S5. Decouple the ladder-type interaction with internal momentum into constant interaction with 2 bond operators.

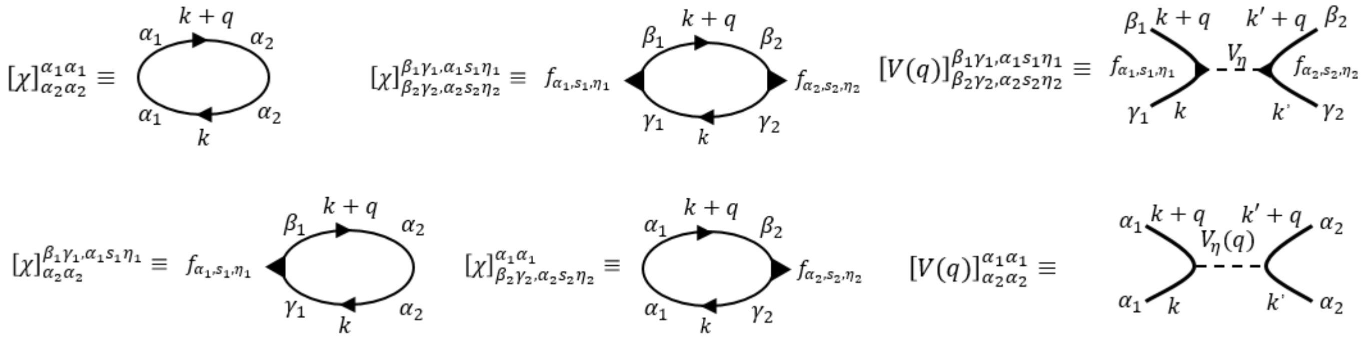

Figure S6. Definition of the susceptibility matrix and vertex indices. The momentum transfer in the form factor is decided by the order of orbital index. When $\epsilon_{\alpha\beta\gamma} = 1$ we have $f_{\alpha,s,\eta} = f_{\alpha,s,\eta}(\boldsymbol{k})$. When $\epsilon_{\alpha\beta\gamma} = -1$, $f_{\alpha,s,\eta}$ represents $f_{\alpha,s,\eta}(\boldsymbol{k}+\boldsymbol{q})$.

Fig.S5. We explicitly show it in the first-order ladder diagram and its formula is given by,

$$
\begin{aligned}
&\sum_{\boldsymbol{k},\boldsymbol{k}'} f_{\alpha_1,s_1,\eta_1}(\boldsymbol{k})G^{\beta_1\beta_2}(\boldsymbol{k}+\boldsymbol{q})G^{\gamma_2\gamma_1}(\boldsymbol{k})V_{\eta_2}(\boldsymbol{k}-\boldsymbol{k}')G^{\beta_2\beta_3}(\boldsymbol{k}'+\boldsymbol{q})G^{\gamma_3\gamma_2}(\boldsymbol{k}')f_{\alpha_3,s_3,\eta_3}(\boldsymbol{k}')\\
&=\sum_{\boldsymbol{k},\boldsymbol{k}',s_2} f_{\alpha_1,s_1,\eta_3)}(\boldsymbol{k})G^{\beta_1\beta_2}(\boldsymbol{k}+\boldsymbol{q})G^{\gamma_2\gamma_1}(\boldsymbol{k})f_{\alpha_2,s_2,\eta_2}(\boldsymbol{k})V_{\eta_2}f_{\alpha_2,s_2,\eta_2}(\boldsymbol{k}')G^{\beta_2\beta_3}(\boldsymbol{k}'+\boldsymbol{q})G^{\gamma_3\gamma_2}(\boldsymbol{k}')f_{\alpha_3,s_3,\eta_3}(\boldsymbol{k}')\\
&=\sum_{s_2}[\chi]^{\beta_1\gamma_1,\alpha_1 s_1\eta_1}_{\beta_2,\alpha_2\gamma_2,s_2\eta_2}(\boldsymbol{q})V_{\eta_2}[\chi]^{\beta_2\gamma_2,\alpha_2 s_2\eta_2}_{\beta_3\gamma_3,\alpha_3 s_3\eta_3}(\boldsymbol{q}).
\end{aligned}
\tag{S26}
$$

Where $G^{\alpha\beta}$ is the Green's function involving sublattice α and β. Thus, a bond susceptibility in a specific channel can be coupled with those in other channels through the ladder diagrams. While, the bubble diagram of bond susceptibilities will introduce a susceptibility in the mixed channel with only one vertex (as shown in Fig.S6), which is the coupling between bond and onsite charge order. Then, the first-order bubble of this mixed susceptibility will involve the onsite susceptibility. The ladder and bubble diagrams couple the onsite and bond charge orders. The full RPA diagrams are displayed in Fig.S7, which can expressed,

$$
[\chi_{RPA}]^{\alpha_1\beta_1,\gamma_1 s_1\eta_1}_{\alpha_2\beta_2,\gamma_2 s_2\eta_2} = [\chi_0]^{\alpha_1\beta_1,\gamma_1 s_1\eta_1}_{\alpha_2\beta_2,\gamma_2 s_2\eta_2} + \sum_{ij,ks\eta}[\chi_0]^{\alpha_1\beta_1,\gamma_1 s_1\eta_1}_{ij,ks\eta}[V_\eta(\boldsymbol{q})]^{ij,ks\eta}_{ij,ks\eta}[\chi_{RPA}]^{ij,ks\eta}_{\alpha_2\beta_2} + [\chi_0]^{\alpha_1\beta_1}_{ii}[V_\eta(\boldsymbol{q})]^{ii}_{jj}[\chi_{RPA}]^{jj}_{\alpha_2\beta_2}, \tag{S27}
$$

$$
[\chi_{RPA}]^{\alpha_1\beta_1,\gamma_1 s_1\eta_1}_{\alpha_2\alpha_2} = [\chi_0]^{\alpha_1\beta_1,\gamma_1 s_1\eta_1}_{\alpha_2\alpha_2} + \sum_{ij,ks\eta}[\chi_0]^{\alpha_1\beta_1}_{ij,s\eta}[V_\eta(\boldsymbol{q})]^{ij,ks\eta}_{ij,ks\eta}[\chi_{RPA}]^{ij,ks\eta}_{\alpha_2\alpha_2} + [\chi_0]^{\alpha_1\beta_1,\gamma_1 s_1\eta_1}_{ii}[V_\eta(\boldsymbol{q})]^{ii}_{jj}[\chi_{RPA}]^{jj}_{\alpha_2\alpha_2}, \tag{S28}
$$

$$
[\chi_{RPA}]^{\alpha_1\alpha_1}_{\alpha_2\alpha_2} = [\chi_0]^{\alpha_1\alpha_1}_{\alpha_2\alpha_2} + \sum_{ij,ks\eta}[\chi_0]^{\alpha_1\alpha_1}_{ij,ks\eta}[V_\eta(\boldsymbol{q})]^{ij,ks\eta}_{ij,ks\eta}[\chi_{RPA}]^{ij,ks\eta}_{\alpha_2\alpha_2} + [\chi_0]^{\alpha_1\alpha_2}_{ii}[V_\eta(\boldsymbol{q})]^{ii}_{jj}[\chi_{RPA}]^{jj}_{\alpha_2\alpha_2}. \tag{S29}
$$

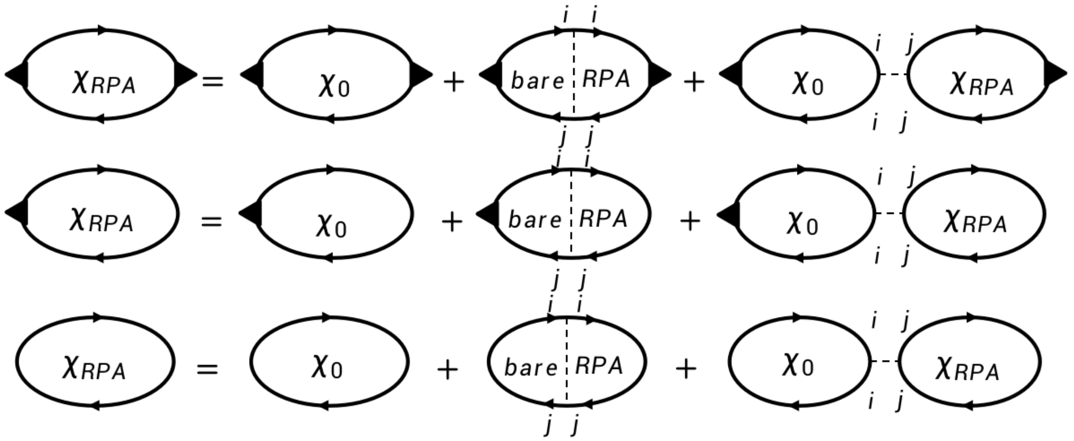

Figure S7. Full RPA diagram for susceptibilities in bond (upper), mixed (middle) and onsite (bottom) channels.

Here, $[\chi]^{\alpha_1\beta_1,\gamma_1 s_1\eta_1}_{\alpha_2\beta_2,\gamma_2 s_2\eta_2}$, $[\chi]^{\alpha_1\beta_1,\gamma_1 s_1\eta_1}_{\alpha_2\alpha_2}$ and $[\chi]^{\alpha_1\alpha_1}_{\alpha_2\alpha_2}$ represent susceptiblity in the bond, mixed and onsite channels, respectively. The upper (lower) indices are those in left (right) part of susceptibility bubble or interaction vertex as shown in Fig.S6. $\alpha, \beta, \gamma \in 1, 2, 3$ are sublattice indices and the $(\gamma s\eta)$ index denotes the form factor. This hierarchy structure motives us to treat oniste and bond charge order together in a RPA matrix form. Under the basis O_o defined in above section, the full 27×27 RPA susceptibility is given by

$$\chi_{RPA}(\boldsymbol{q}) = (I + \chi_0(\boldsymbol{q})\mathcal{U}_c(\boldsymbol{q}))^{-1}\chi_0(\boldsymbol{q}), \tag{S30}$$

where the interaction matrix reads,

$$\begin{aligned} \mathcal{U}_c(\boldsymbol{q}) &= \begin{pmatrix} V_1^c & 0 & 0 \\ 0 & V_2^c & 0 \\ 0 & 0 & U^c(\boldsymbol{q}) \end{pmatrix}, \\ U^c(q) &= \begin{pmatrix} 0 & V_{12} & V_{13} \\ V_{12} & 0 & V_{23} \\ V_{13} & V_{23} & 0 \end{pmatrix}, \\ V_\eta^c &= -\operatorname{diag}\left\{2V_\eta, 2V_\eta, \dots\right\}, \\ V_{\beta\gamma} &= \sum_\eta 2V_\eta f_{\alpha,+,\eta}(\boldsymbol{q}). \end{aligned} \tag{S31}$$

Here V_η^c is a 12×12 matrix for the η-th bond channel.

IV. CLASSIC ENERGY FOR THE CDW WITH INTRA-UNIT CHARGE MODULATION

In this section, we discuss the classical potential energy gain of the nSDM state, which mainly involves charge density modulation within three sublattices. Assuming the charge density in three sublattices is modified to $n_1 = n + (\delta_2 + \delta_3),\ n_2 = n - \delta_2,\ n_3 = n - \delta_3$, where n is the normal charge density, we discussion the potential energy gain under different inter-site repulsion.

Under the NN repulsion V_1, the energy difference relative to the normal state is

$$\Delta E_{V_1} = 2V_1(n_1 n_2 + n_2 n_3 + n_3 n_1 - 3n^2) = -2V_1(\delta_2\delta_3 + \delta_2^2 + \delta_3^2) < 0. \tag{S32}$$

Under the NNN repulsion V_2, the energy difference relative to the normal state is

$$\Delta E_{V_2} = 2V_2(n_1 n_2 + n_2 n_3 + n_3 n_1 - 3n^2) = -2V_2(\delta_2\delta_3 + \delta_2^2 + \delta_3^2) < 0. \tag{S33}$$

Under the NNNN repulsion V_3, which is a intra-sublattice repulsion, the energy gain is

$$\Delta E_{V_3} = 3V_3(n_1^2 + n_2^2 + n_3^2 - 3n^2) = 3V_3[(\delta_2 + \delta_3)^2 + \delta_2^2 + \delta_3^2] > 0. \tag{S34}$$

From the above expressions, we can conclude that V_1 and V_2 will promote the nSDM phase while V_3 will suppress it. The energy gain is increases linearly with the replsion $V_{1,2}$ and this explains that nSDM is favored when the repulsion is strong.

V. PHYSICAL UNDERSTANDING OF CDW ORDER TENDENCY FOR NN AND NNN BONDS

In this section, we provide physical understanding about the favored orders on the NN and NNN bonds, using weak-coupling analysis. There are three Fermi surface nesting vectors $\boldsymbol{Q}_{1,2,3}$ connecting three pairs of opposite edges. The charge density wave orders with $\boldsymbol{Q}_{1,2,3}$ are in general degenerate and thus we can choose one charge order with $\boldsymbol{Q}_i$ to study its effect on the band structures. Taking the charge order with $\boldsymbol{Q}_1$ as an example, the effective Hamiltonian in the band space with the inclusion of this CDW reads,

$$H_{\rm eff}(\boldsymbol{k}) = \begin{pmatrix} \epsilon_\nu(\boldsymbol{k}) & \Delta(\boldsymbol{k}) \\ \Delta^\dagger(\boldsymbol{k}) & \epsilon_\nu(\boldsymbol{k}+\boldsymbol{Q}_1) \end{pmatrix}, \tag{S35}$$

where the $\epsilon_\nu(\boldsymbol{k})$ is the ν-th band energy and $\Delta(\boldsymbol{k})$ is the CDW order parameter in the band space. In the weak-coupling limit ($\Delta(\boldsymbol{k})$ is small), the gap opening is prominent around the Fermi level, i.e. $\epsilon_\nu(\boldsymbol{k}) \approx 0$, and the relevant states are the Fermi segments connected by the $\boldsymbol{Q}_1$ (shown in Fig.1(b)). The instability of this CDW order is determined by the energy gain, which is directly proportional to the induced gap size on the Fermi surface. Thus, we can explore the gap size on the Fermi surfaces of various CDW orders to examine the CDW formation propensity. In the following, we study both onsite and bond CDW orders in the band space.

The onsite CDW operator is $n_\alpha(\boldsymbol{q}) = \frac{1}{\sqrt{N}}\sum_{\boldsymbol{k}} c^\dagger_\alpha(\boldsymbol{k}+\boldsymbol{q})c_\alpha(\boldsymbol{k})$. The corresponding CDW order parameter in the band space reads $\Delta^\nu_{n_\alpha}(\boldsymbol{k}) \propto \langle c^\dagger_\alpha(\boldsymbol{k}+\boldsymbol{Q}_1)c_\alpha(\boldsymbol{k})\rangle_\nu$, where $\langle\rangle_\nu$ is the average over the eigenvector of the ν-th band. Due to the sublattice texture on the Fermi surface, $\Delta^\nu_n(\boldsymbol{k})$ is zero when $\boldsymbol{k}$ is located at the VHS and reaches the maximum at the midpoints between two VHSs. Thus, the VHSs with large DOS cannot be gapped by this onsite CDW order and this leads to a small energy gain.

The bond CDW operator on the $\beta\gamma$ bond is $B_{\alpha,\pm,\eta}(\boldsymbol{q}) = \frac{1}{\sqrt{N}}\sum_{\boldsymbol{k}} \tilde{f}_{\alpha,\pm,\eta}(\boldsymbol{k})c^\dagger_\beta(\boldsymbol{k}+\boldsymbol{q})c_\gamma(\boldsymbol{k})$. The real and imaginary bond order operators in the symmetric and anti-symmetric channels are given by,

$$B'^{('')}_{\alpha,\pm,\eta}(\boldsymbol{q}) = \frac{1}{2(i)}\{\tilde{B}_{\alpha,\pm,\eta}(\boldsymbol{q}) \pm [\tilde{B}_{\alpha,\pm,\eta}(\boldsymbol{q})]^\dagger\}, \tag{S36}$$

$$= \frac{1}{2(i)}\frac{1}{\sqrt{N}}\sum_{\boldsymbol{k}}[\tilde{f}_{\alpha,\pm,\eta}(\boldsymbol{k})c^\dagger_\beta(\boldsymbol{k}+\boldsymbol{q})c_\gamma(\boldsymbol{k}) \pm \tilde{f}^*_{\alpha,\pm,\eta}(\boldsymbol{k})c^\dagger_\gamma(\boldsymbol{k})c_\beta(\boldsymbol{k}-\boldsymbol{q})] \tag{S37}$$

Setting $\boldsymbol{q} = \boldsymbol{Q}_1$ and $(\alpha\beta\gamma) = (123)$, these operators can be further written as

$$B'^{('')}_{\alpha,\pm,\eta}(\boldsymbol{Q}_1) = \frac{1}{2(i)}\frac{1}{\sqrt{N}}\sum_{\boldsymbol{k}}[-\tilde{f}_{\alpha,\pm,\eta}(\boldsymbol{k}-\boldsymbol{Q}_1)c^\dagger_\beta(\boldsymbol{k})c_\gamma(\boldsymbol{k}+\boldsymbol{Q}_1) \mp \tilde{f}^*_{\alpha,\pm,\eta}(\boldsymbol{k})c^\dagger_\gamma(\boldsymbol{k})c_\beta(\boldsymbol{k}+\boldsymbol{Q}_1)]. \tag{S38}$$

Due to $\boldsymbol{Q}_1 \cdot \boldsymbol{l}_{1,\rm NN} = \pi$ and $\boldsymbol{Q}_1 \cdot \boldsymbol{l}_{1,\rm NNN} = 0$, the operators on the NN and NNN bonds can be written as,

$$B'^{('')}_{\alpha,\pm,\rm NN}(\boldsymbol{Q}_1) = \frac{1}{2(i)}\frac{1}{\sqrt{N}}\sum_{\boldsymbol{k}}[\tilde{f}_{\alpha,\pm,\rm NN}(\boldsymbol{k})c^\dagger_\beta(\boldsymbol{k})c_\gamma(\boldsymbol{k}+\boldsymbol{Q}_1) \mp \tilde{f}^*_{\alpha,\pm,\rm NN}(\boldsymbol{k})c^\dagger_\gamma(\boldsymbol{k})c_\beta(\boldsymbol{k}+\boldsymbol{Q}_1)], \tag{S39}$$

$$B'^{('')}_{\alpha,\pm,\rm NNN}(\boldsymbol{Q}_1) = \frac{1}{2(i)}\frac{1}{\sqrt{N}}\sum_{\boldsymbol{k}}[-\tilde{f}_{\alpha,\pm,\rm NNN}(\boldsymbol{k})c^\dagger_\beta(\boldsymbol{k})c_\gamma(\boldsymbol{k}+\boldsymbol{Q}_1) \mp \tilde{f}^*_{\alpha,\pm,\rm NNN}(\boldsymbol{k})c^\dagger_\gamma(\boldsymbol{k})c_\beta(\boldsymbol{k}+\boldsymbol{Q}_1)]. \tag{S40}$$

On the two Fermi surface segments, we have $\boldsymbol{k}\cdot\boldsymbol{l}_{\alpha,\rm NN} = \pm\pi/2$ and $\boldsymbol{k}_{\beta,\gamma}\cdot\boldsymbol{l}_{\alpha,\rm NNN} = \pm\pi/2$. The NN and NNN symmetric form factors vanish on the VHSs and thus the corresponding bond CDW order parameter $\Delta^\nu_B(\boldsymbol{k})$ on the Fermi surface is small, making them less favored. In the anti-symmetric channel, the form factor is imaginary and we have $\tilde{f}^*_{\alpha,-,\eta}(\boldsymbol{k}) = -\tilde{f}_{\alpha,-,\eta}(\boldsymbol{k})$. The anti-symmetric CDW order parameters on the NN and NN bonds can be rewritten as,

$$\Delta^\nu_{B'/''_{NN}}(\boldsymbol{k}) \propto \tilde{f}_{\alpha,-,\rm NN}(\boldsymbol{k})\langle c^\dagger_\beta(\boldsymbol{k})c_\gamma(\boldsymbol{k}+\boldsymbol{Q}_1) \pm c^\dagger_\gamma(\boldsymbol{k})c_\beta(\boldsymbol{k}+\boldsymbol{Q}_1)\rangle_\nu, \tag{S41}$$

$$\Delta^\nu_{B'/''_{NNN}}(\boldsymbol{k}) \propto -\tilde{f}_{\alpha,-,\rm NNN}(\boldsymbol{k})\langle c^\dagger_\beta(\boldsymbol{k})c_\gamma(\boldsymbol{k}+\boldsymbol{Q}_1) \mp c^\dagger_\gamma(\boldsymbol{k})c_\beta(\boldsymbol{k}+\boldsymbol{Q}_1)\rangle_\nu. \tag{S42}$$

For the NN bond order parameter, $\tilde{f}_{\alpha,-,\rm NN}(\boldsymbol{k}) = i$ for $\boldsymbol{k}$ on the Fermi surface segments. When $\boldsymbol{k}$ is around the VHSs, the CDW order parameters $\Delta^\nu_{B'/''_{NN}}(\boldsymbol{k})$ are close in both channels due to the pure sublattice feature. When $\boldsymbol{k}$ moves away from the VHSs, the

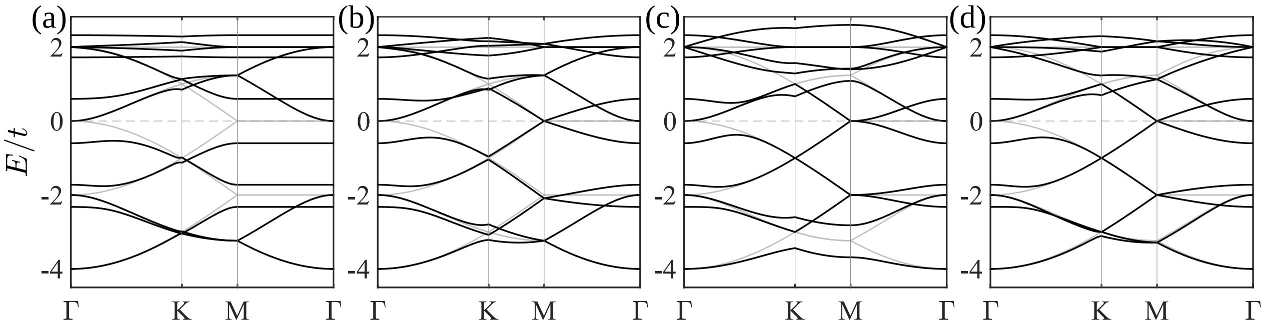

Figure S8. Band structure with bond CDW on the NN and NNN bonds: 1Q real (a) and imaginary (b) bond order on the NN bond; 1Q real (a) and imaginary (b) bond order on the NNN bond. The gray and black curves denote the folded bands without and with CDW order. Here the bond order parameters are the same for four plots.

corresponding eigenstate is a mixture of two sublattices. This leads to enhanced $\Delta^{\nu}_{B'_{NN}}(\boldsymbol{k})$ but decreased $\Delta^{\nu}_{B''_{NN}}(\boldsymbol{k})$ due to the addition and cancelation between two terms. Especially, $\Delta^{\nu}_{B''_{NN}}(\boldsymbol{k})$ vanishes at the midpoint between two VHSs. Therefore, the real bond CDW is more favorable than the imaginary CDW in the anti-symmetric NN channel. For the NNN bond orders, $\tilde{f}_{\alpha,-,\mathrm{NN}}(\boldsymbol{k})$ varies with $\boldsymbol{k}$ and drops to zero at the midpoint between two VHSs. The behaviors of $\Delta^{\nu}_{B''_{NNN}}(\boldsymbol{k})$ is the opposite with those of $\Delta^{\nu}_{B''_{NN}}(\boldsymbol{k})$ according to Eq.S42. Thus, the imaginary bond CDW is more favorable than the real CDW in the anti-symmetric NNN channel. These insights provide a physical understanding for our analysis of relative CDW fluctuations and CDW order tendency on these bonds in the main text. We further perform numerical calculations with different CDW orders, as shown in Fig.S8. From the case of bond order on the NN bond (a) (real) and (b) (imaginary), it is evident that the real bond order open large CDW gap on the Fermi surface. Similarly, the CDW gap for real and imaginary bond orders are comparable but around the M point, the CDW gap of the imaginary bond order is larger. All these results are consistent with our above analytical analysis.

VI. CHARGE PATTERNS OF CDW ORDER AND THEIR IMPACT ON BAND STRUCTURES

In this section, we study the CDW pattern of CDW instabilities and their impact on the electronic structures. With decreasing temperature, an eigenvalue of the RPA susceptibility χ_{RPA} at Γ or $\mathbf{M}$ diverges, signaling an instability at this vector. The CDW pattern is associated with the corresponding eigenvector. Fig.S9 shows the largest eigenvalues of the susceptibility matrix along the high-symmetry path with different interactions at two temperatures. The soild and dashed lines denote 1st and 2nd largest eigenvalue, respectively, while the arrows show the corresponding CDW pattern at Γ or $\mathbf{M}$ points. These results fully agree with our calculations presented in Fig.2 in the main text.

We further study the effect of these orders on the band structures. Within a CDW state, its effect can be described by the symmetry-breaking mean-field Hamiltonian,

$$\begin{aligned} H^{\mathrm{MF}}_{\mathrm{CDW}} = \sum_{R,\alpha} [& (t + \Delta^{Re}_{\alpha,+,NN}(R) + i\Delta^{Im}_{\alpha,+,NN}(R) + \Delta^{Re}_{\alpha,-,NN}(R) + i\Delta^{Im}_{\alpha,-,NN}(R)) c^{\dagger}_{\beta,r} c_{\gamma,r+a_\alpha/2} \\ & + (t + \Delta^{Re}_{\alpha,+,NN}(R) + i\Delta^{Im}_{\alpha,+,NN}(R) - \Delta^{Re}_{\alpha,-,NN}(R) - i\Delta^{Im}_{\alpha,-,NN}(R)) c^{\dagger}_{\beta,r} c_{\gamma,r-a_\alpha/2} \\ & + (\Delta^{Re}_{\alpha,+,NNN}(R) + i\Delta^{Im}_{\alpha,+,NNN}(R) + \Delta^{Re}_{\alpha,-,NNN}(R) + i\Delta^{Im}_{\alpha,-,NNN}(R)) c^{\dagger}_{\beta,r} c_{\gamma,r+(a_\beta-a_\gamma)/2} \\ & + (\Delta^{Re}_{\alpha,+,NNN}(R) + i\Delta^{Im}_{\alpha,+,NNN}(R) - \Delta^{Re}_{\alpha,-,NNN}(R) - i\Delta^{Im}_{\alpha,-,NNN}(R)) c^{\dagger}_{\beta,r} c_{\gamma,r-(a_\beta-a_\gamma)/2} - (\mu + \rho_\alpha(R)) c^{\dagger}_{r,\alpha} c_{r,\alpha}]. \end{aligned} \tag{S43}$$

Here, R is the coordinate of the unicell, r is the coordinate of the corresponding sublattice, $t = -1$ is the hopping integral and Δ is the CDW order parameter. $\Delta^{Re/Im}_{\alpha,\pm,\eta}(R) = \Delta^{Re/Im}_{\alpha,\pm,\eta}\cos(\boldsymbol{Q}\cdot\boldsymbol{R})$ is the order parameter of the symmetric or antisymmetric CBO/LCO, $\rho_\alpha(R) = \rho_\alpha\cos(\boldsymbol{Q}\cdot\boldsymbol{R})$ is the onsite charge order and μ is the chemical potential at the p-type VH filling ($\mu = 0$).

We show some representative CDW patterns from the eigenvector of RPA susceptibility. In the V_1-dominant regime, the real bond fluctuation at M point is strong, as shown in Fig.S9(b), and the CDW pattern mainly involves NN and NNN bonds. The corresponding order parameters are $\Delta^{Re}_{\alpha,-,NN} : \Delta^{Re}_{\alpha,-,NNN} \approx -0.0604 : 0.0366$. In the V_2-dominant regime, the imaginary bond fluctuation at M point is strong, as shown in Fig.S9(c), and the CDW pattern mainly involves NN and NNN bonds. The corresponding

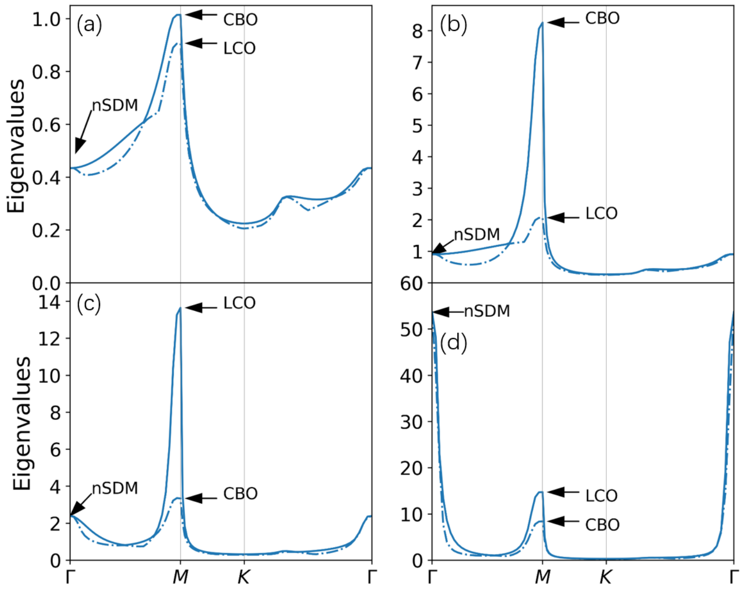

Figure S9. Eigenvalue of susceptibility matrix along high symmetry line. (a) Bare susceptibility (b) $V_1 = 0.6$, $V_2 = 0.0$. (c) $V_1 = 0.0$, $V_2 = 0.95$. (d) $V_1 = 0.5$, $V_2 = 0.75$. The temperatures in (a)-(c) and (d) are $\beta = 200$ and $\beta = 100$, respectively, same parameter with Fig2 in main text. Solid line and dashed line are 1st and 2nd largest eigenvalue.

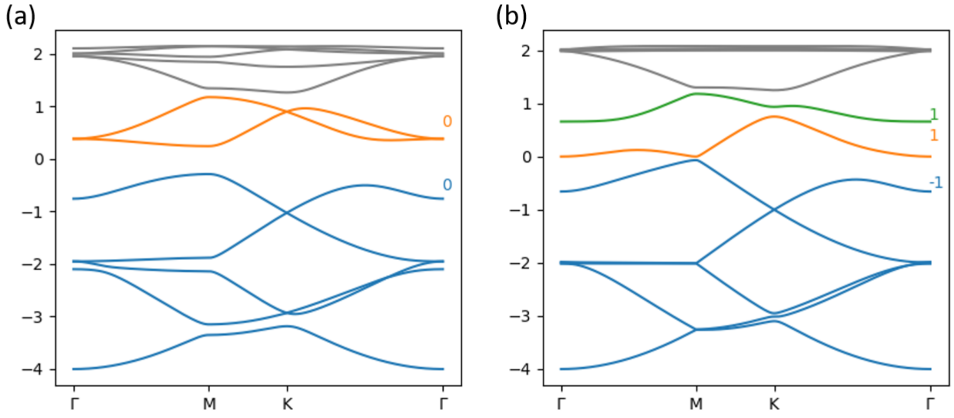

Figure S10. Energy band of CBO and LCO phase in folded BZ, numbers near the bands with same color are corresponding Chern number of the bands

order parameters are $\Delta^{Im}_{\alpha,-,NN} : \Delta^{Im}_{\alpha,-,NNN} \approx -0.0469 : 0.0528$. In both regimes, the 3Q will be more energetically favored according to our analysis. With strong repulsion, onsite CDW fluctuation at Γ point is dominant and the corresponding CDW pattern involving symmetric bond fluctuations is two-fold. The two-fold order parameters are: $\rho_1 : \rho_2 : \rho_3 = 2 : -1 : -1$, $\Delta_{2,+,NN} = \Delta_{3,+,NNN}$ and $\rho_1 : \rho_2 : \rho_3 = 0 : 1 : -1$, $\Delta_{2,+,NN} = -\Delta_{3,+,NNN}$. According to our analysis in the main text, this state forms a nematic order, breaking the six-fold rotational symmetry.

In the main text, we show the unfolded band structure in 3Q CBO and 3Q LCO and nSDM states in Fig.4 of the main text. The

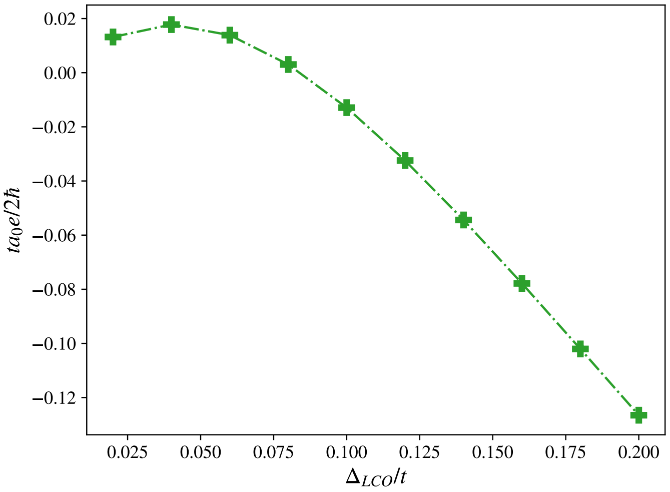

Figure S11. Orbital maganzation with LCO $1.5\Delta_{LCO} = 1.5\Delta_{LCO}^{NN} = -\Delta_{LCO}^{NNN}$ per 2×2 supercell.

adopted order parameters used are (a) $\Delta^{Re}_{\alpha,-,NN} = -0.12, \Delta^{Re}_{\alpha,-,NNN} = 0.07$ for CBO phase, (b) $\Delta^{Im}_{\alpha,-,NN} = -0.09, \Delta^{Im}_{\alpha,-,NNN} = 0.10$ for LCO phase and (c) $\rho_1 = 0.2, \rho_2 = \rho_3 = -0.1$, $\Delta^{Re}_{2,+,NN} = \Delta^{Re}_{3,+,NN} = 0.05$ for nSDM phase. The corresponding folded band structures of 3Q CBO and LCO phase are displayed in Fig.S10. The CBO opens a isotropic full gap around the Fermi energy. For the LCO phase, the gap is nearly closed at the M point folded Brillouin zone. Since the LCO phase breaks the time reversal symmetry, which introduces non-trivial Chern numbers in band structures, shown in Fig.S10. The total Chern number for the occupied bands is -1 in the LCO phase. The nonzero total Chern number in LCO phase can lead to an anomalous hall effect and the LCO can generate orbital magnetism, which may make it possible to distinguish CBO and LCO in experiment.

VII. ORBITAL MAGNETIC MOMENT OF THE LOOP CURRENT ORDER

There is no local moment in the loop current phase since there is no net flux. However, as this loop current order spontaneously breaks the time-reversal symmetry. It still lead to a non-zero orbital magnetization, which is given by[S2]

$$\boldsymbol{M} = \sum_n \int_{BZ} \frac{d\boldsymbol{k}}{(2\pi)^2}[\boldsymbol{m}_n(\boldsymbol{k}) - \frac{e}{\hbar}\epsilon_{n\boldsymbol{k}}\Omega_n(\boldsymbol{k})]f(\epsilon_{n\boldsymbol{k}}). \tag{S44}$$

$\boldsymbol{m}_n(\boldsymbol{k})$ is the orbital moment of n-th band and $\Omega_n(\boldsymbol{k})$ is the berry curvature. They are given by

$$\boldsymbol{m}_{n\boldsymbol{k}} = -i\frac{e}{2\hbar}(\langle\frac{\partial u_{nk}}{\partial k_x}|(H_{\boldsymbol{k}} - \epsilon_{nk})|\frac{\partial u_{nk}}{\partial k_y}\rangle - H.c.)\hat{z} \tag{S45}$$

and

$$\Omega_n(\boldsymbol{k}) = i(\langle\frac{\partial u_{nk}}{\partial k_x}|\frac{\partial u_{nk}}{\partial k_y}\rangle - H.c.). \tag{S46}$$

The numerical result of orbital magnetization is shown in Fig.S11. In CsV_3Sb_5 we use lattice constant $a_0 \simeq 5.4$Å and $t = 0.5$eV, then the unit $\frac{ta_0^2 e}{2\hbar} \simeq 1.9\mu_B$. Then the total magnetization is about $10^{-2}\mu_B$.

VIII. EFFECT OF NEXT-NEAREST HOPPING

In the real material, next-nearest hopping t' will be non-zero. In our model, a finite t' will change the shape of the Fermi surface, and suppress the perfect nesting at M point as shown in Fig.S12. However, the LCO can still be the leading instability under suitable

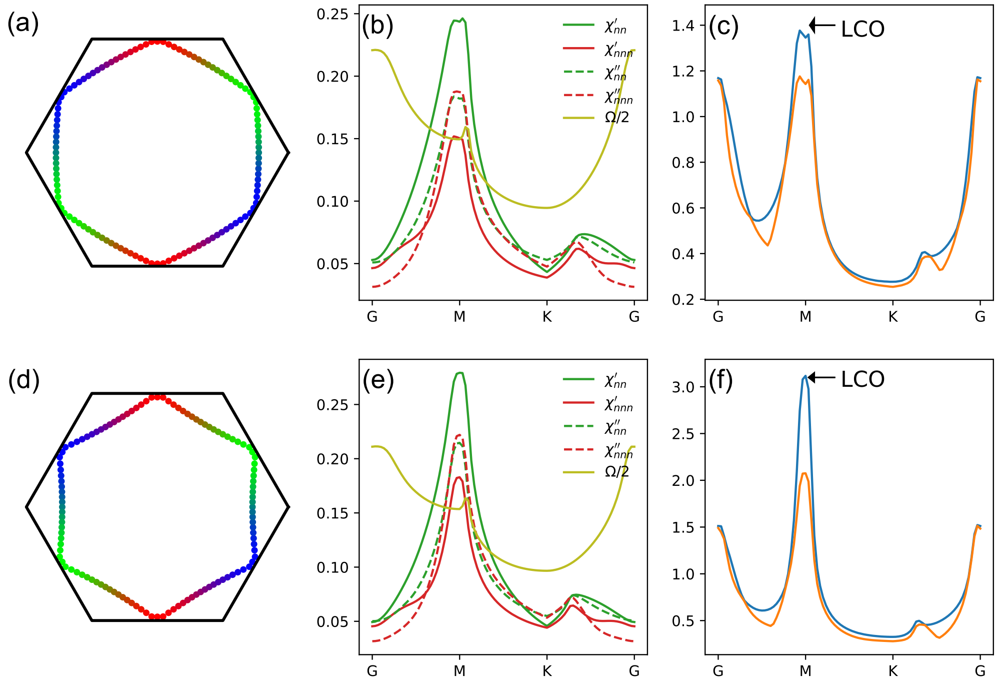

Figure S12. Fermi surface and susceptibilities with finite t' (a)-(c) $t' = 0.05$ (d)-(f) $t' = -0.05$. (a) and (c) are the fermi surfaces. (b) and (e) are bare susceptibilities along the high-symmetry line. (c) and (f) are the first two largest eigenvalues under interaction (c): $V_1 = 0.0$ $V_2 = 0.7$, (d): $V_1 = 0.0$ $V_2 = 0.85$. Susceptibilities are calculated under $\beta = 200$

interaction. Our calculation shows that when t' is negative, the Fermi surface turns to a shrinked hexagon. This shape will make the nesting vector between points aside to two VHSs closer to **M**, which compensates the violation of perfect nesting and makes the **M** point susceptibility peak more stable. In the real 135 kagome material the Fermi surface is closer to the negative t' case. It leads to a robust loop current phase. As shown in Fig.S13, this LCO phase can survive in $t' = -0.1$.

IX. EFFECTIVE PAIRING INTERACTION FROM ONSITE AND BOND CHARGE FLUCTUATIONS

When the Fermi level moves away from VHSs, the Fermi surface nesting weakens, leading to the suppression of both onsite and bond charge orders. However, these charge fluctuations can promote particle-particle instabilities, i.e. superconductivity. In this section, we study the superconducting pairing from these fluctuations of both onsite and bond charge orders based the Feynman diagrams. In the sublattice space, the general effective Cooper pair scattering interaction is

$$V^{\text{eff}} = \sum_{pqst} \tilde{\Gamma}^{pq}_{st}(\boldsymbol{k}, \boldsymbol{k'}) c^\dagger_p(\boldsymbol{k}) c^\dagger_q(-\boldsymbol{k}) c_s(-\boldsymbol{k'}) c_t(\boldsymbol{k'}), \tag{S47}$$

where p, q, s, t are the sublattice indices and the $\tilde{\Gamma}^{pq}_{st}(\boldsymbol{k}, \boldsymbol{k'})$ is anti-symmetric in the spinless case. In the following, we deal with the effective interactions using the conventional diagrams technique and then do the antisymmetrization to obtain effective interaction for the spinless case. The bare interaction can be written as in a compact form

$$H_I = \frac{1}{N} \sum_{\gamma,\eta} \sum_{\boldsymbol{k},\boldsymbol{k'},\boldsymbol{q}} V_{\gamma,\eta}(\boldsymbol{q}) c^\dagger_\alpha(\boldsymbol{k}) c_\alpha(\boldsymbol{k}+\boldsymbol{q}) c^\dagger_\beta(\boldsymbol{k'}+\boldsymbol{q}) c_\beta(\boldsymbol{k'}), \tag{S48}$$

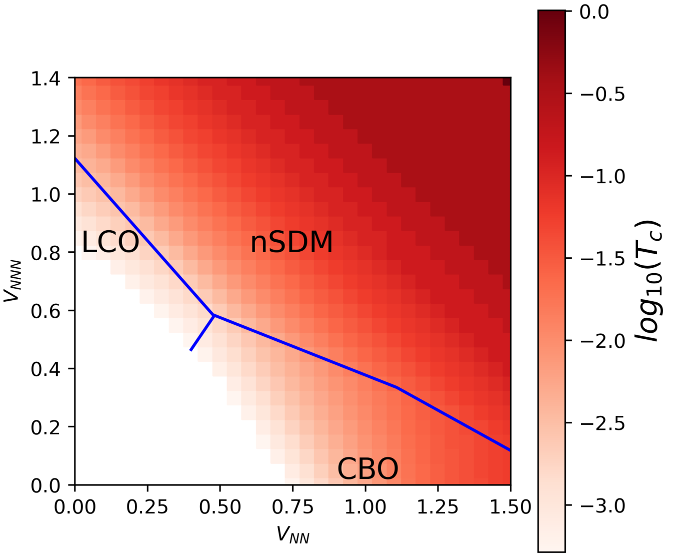

Figure S13. RPA phase diagram of charge orders when $t' = -0.1$.

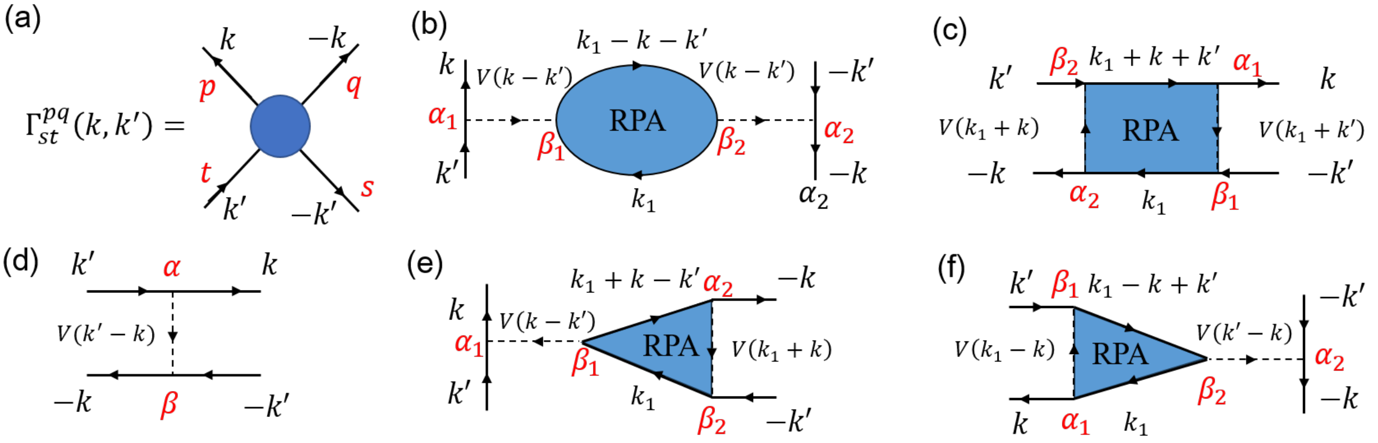

Figure S14. Superconductivity pairing given under RPA approximation. (a) Definition of the pairing kernel Γ^{pq}_{st}. (d) effective pairing at tree level. (b)(c)(e)(f) 2nd order Effective interaction vertex in SC pairing (a) bubble vertex (b) ladder vertex (c-d) vertex corrections. Red characters are sublattice indices.

with α, β, γ being the sublattice indices and $\eta = 1, 2$ represents NN or NNN repulsion. We have $V_{\gamma,\eta}(\boldsymbol{q}) = V_{\gamma,\eta}\cos(\boldsymbol{q}\cdot l_{\gamma,\eta})$ and $V_{1,\eta} = V_{2,\eta} = V_{3,\eta} = V_\eta$ for normal isotropic Colomb repulsion.

All the diagrams that contribute to the effective interactions are displayed in Fig.S14(b-f).The effective pairing interaction from the first-order diagrams reads,

$$\Gamma^{\alpha\beta(1)}_{\beta\alpha}(\boldsymbol{k},\boldsymbol{k'}) = \Gamma^{\beta\alpha(1)}_{\alpha\beta}(\boldsymbol{k},\boldsymbol{k'}) = V_{\gamma,1}\cos(\boldsymbol{q}\cdot\boldsymbol{l_{\gamma,1}}) + V_{\gamma,2}\cos(\boldsymbol{q}\cdot\boldsymbol{l_{\gamma,2}}) \equiv V_\gamma(\boldsymbol{q}) = V(\boldsymbol{q}),$$

with $\boldsymbol{q} = \boldsymbol{k} - \boldsymbol{k'}$. At second-order both bubble and ladder diagrams get involved. Here the pairing interaction is mediated by

fluctuations in the onsite, bond and mixed channels. For the bubble diagram shown in Fig.S14 (b), the effective interaction comes from onsite charge fluctuations, which is given by,

$$\Gamma^{\alpha_1\alpha_2}_{\alpha_2\alpha_1}(\boldsymbol{k},\boldsymbol{k}') = V_{\gamma_1}(\boldsymbol{q})[\chi(\boldsymbol{q})]^{\beta_1\beta_1}_{\beta_2\beta_2}V_{\gamma_2}(\boldsymbol{q}).$$

For the vertex correction (VC) and ladder diagrams, we need to choose suitable decoupling for the internal-momentum dependent interaction, based on the indices of the vertices. For the VC diagram in Fig.S14 (e), the interaction reads,

$$\Gamma^{\alpha_1\alpha_2}_{\beta_2\alpha_1}(\boldsymbol{k},\boldsymbol{k}') = \begin{cases} \sum_{s_2,\eta_2} s_2 V_{\gamma_1}(\boldsymbol{q})[\chi(\boldsymbol{q})]^{\beta_1\beta_1}_{\alpha_2\beta_2,\gamma_2 s_2\eta_2} V_{\gamma_2,\eta_2} f_{\gamma_2,s_2,\eta_2}(\boldsymbol{k}) & \epsilon_{\alpha_2\beta_2\gamma_2} = 1 \\ \sum_{s_2,\eta_2} V_{\gamma_1}(\boldsymbol{q})[\chi(\boldsymbol{q})]^{\beta_1\beta_1}_{\alpha_2\beta_2,\gamma_2 s_2\eta_2} V_{\gamma_2,\eta_2} f_{\gamma_2,s_2,\eta_2}(\boldsymbol{k}') & \epsilon_{\alpha_2,\beta_2,\gamma_2} = -1 \end{cases} \tag{S49}$$

The relevant indices are provided in VC in FigS14 (e). Similarly, for the VC diagram in FigS14 (f), the interaction reads,

$$\Gamma^{\alpha_1\alpha_2}_{\alpha_2\beta_1}(\boldsymbol{k},\boldsymbol{k}') = \begin{cases} \sum_{s_1,\eta_1} s_1 V_{\gamma_1,\eta_1} f_{\gamma_1,s_1,\eta_1}(\boldsymbol{k})[\chi(\boldsymbol{q})]^{\beta_1\alpha_1,\gamma_1 s_1\eta_1}_{\beta_2\beta_2} V_{\gamma_2}(\boldsymbol{q}) & \epsilon_{\beta_1,\alpha_1,\gamma_1} = 1, \\ \sum_{s_1,\eta_1} V_{\gamma_1,\eta_1} f_{\gamma_1,s_1,\eta_1}(\boldsymbol{k}')[\chi(\boldsymbol{q})]^{\beta_1\alpha_1,\gamma_1\eta_1}_{\beta_2\beta_2} V_{\gamma_2}(\boldsymbol{q}) & \epsilon_{\beta_1,\alpha_1,\gamma_1} = -1 \end{cases} \tag{S50}$$

According our calculations, we find the these VC diagrams are numerically small but nonzero.

For the ladder diagram shown in Fig.S14 (c), the interaction reads

$$\Gamma^{\alpha_1\alpha_2}_{\beta_1\beta_2}(\boldsymbol{k},\boldsymbol{k}') = \begin{cases} \sum_{s_1,s_2,\eta_1,\eta_2} V_{\gamma_1,\eta_1} V_{\gamma_2,\eta_2}(s_1 s_2 f_{\gamma_2,s_2,\eta_2}(\boldsymbol{k})[\chi(\boldsymbol{q}')]^{\beta_2\alpha_2,\gamma_2 s_2\eta_2}_{\alpha_1\beta_1,\gamma_1 s_1\eta_1} f_{\gamma_1,s_1,\eta_1}(\boldsymbol{k}')) & \epsilon_{\beta_2,\alpha_2,\gamma_2} = 1,\ \epsilon_{\alpha_1,\beta_1,\gamma_1} = 1 \\ \sum_{s_1,s_2,\eta_1,\eta_2} V_{\gamma_1,\eta_1} V_{\gamma_2,\eta_2}(s_1 f_{\gamma_2,s_2,\eta_2}(\boldsymbol{k})[\chi(\boldsymbol{q}')]^{\beta_2\alpha_2,\gamma_2 s_2\eta_2}_{\alpha_1\beta_1,\gamma_1 s_1\eta_1} f_{\gamma_1,s_1,\eta_1}(\boldsymbol{k})) & \epsilon_{\beta_2,\alpha_2,\gamma_2} = 1,\ \epsilon_{\alpha_1,\beta_1,\gamma_1} = -1 \\ \sum_{s_1,s_2,\eta_1,\eta_2} V_{\gamma_1,\eta_1} V_{\gamma_2,\eta_2}(s_2 f_{\gamma_2,s_2,\eta_2}(\boldsymbol{k}')[\chi(\boldsymbol{q}')]^{\beta_2\alpha_2,\gamma_2 s_2\eta_2}_{\alpha_1\beta_1,\gamma_1 s_1\eta_1} f_{\gamma_1,s_1,\eta_1}(\boldsymbol{k})) & \epsilon_{\beta_2,\alpha_2,\gamma_2} = -1,\ \epsilon_{\alpha_1,\beta_1,\gamma_1} = 1 \\ \sum_{s_1,s_2,\eta_1,\eta_2} V_{\gamma_1,\eta_1} V_{\gamma_2,\eta_2}(f_{\gamma_2,s_2,\eta_2}(\boldsymbol{k}')[\chi(\boldsymbol{q}')]^{\beta_2\alpha_2,\gamma_2 s_2\eta_2}_{\alpha_1\beta_1,\gamma_1 s_1\eta_1} f_{\gamma_1,s_1,\eta_1}(\boldsymbol{k})) & \epsilon_{\beta_2,\alpha_2,\gamma_2} = -1,\ \epsilon_{\alpha_1,\beta_1,\gamma_1} = -1 \end{cases} \tag{S51}$$

with $\boldsymbol{q}' = \boldsymbol{k} + \boldsymbol{k}'$ in above equations. Among above cases $\epsilon_{\alpha_a,\beta_a,\gamma_a} = 0$ is not allowed. By projecting the effective interaction onto the Fermi surface, the pairing vertex in the band space is given by [S3–S6],

$$\Gamma_{\mu\nu}(\boldsymbol{k},\boldsymbol{k}') = \sum_{pqst} \Gamma^{pq}_{st}(k,k') a^{p*}_{\mu}(\boldsymbol{k}) a^{q*}_{\mu}(-\boldsymbol{k}) a^{s}_{\nu}(-\boldsymbol{k}') a^{t}_{\nu}(\boldsymbol{k}'), \tag{S52}$$

where $a^{q}_{\mu}(\boldsymbol{k})$ is the eigen state on the μ band at the Fermi point $\boldsymbol{k}$. The pairing vertex in our spinless case can be obtained by performing the anti-symmetrization on this effective interaction,

$$\tilde{\Gamma}(\boldsymbol{k},\boldsymbol{k}') = \Gamma(\boldsymbol{k},\boldsymbol{k}') - \Gamma(\boldsymbol{k},-\boldsymbol{k}') \tag{S53}$$

Near the transition temperature T_c, the gap function can be obtained by solving the following linearized gap equation,

$$-\int_{\mathrm{FS}} \frac{d\boldsymbol{k}'}{V_G\,|v_F(\boldsymbol{k}')|} \tilde{\Gamma}(\boldsymbol{k},\boldsymbol{k}')\,\Delta(\boldsymbol{k}') = \lambda\Delta(\boldsymbol{k}), \tag{S54}$$

where where $v_F(\boldsymbol{k}')$ is the Fermi velocity at the momentum $\boldsymbol{k}'$ on the Fermi surface and $V_G = \frac{8\pi^2}{\sqrt{3}}$ is the area the of Brillouin zone. Here λ is the pairing strength and $\Delta(\boldsymbol{k})$ is the corresponding gap function on the Fermi surface. The dominant pairing is represented by the gap function associated with the largest positive pairing eigenvalue.

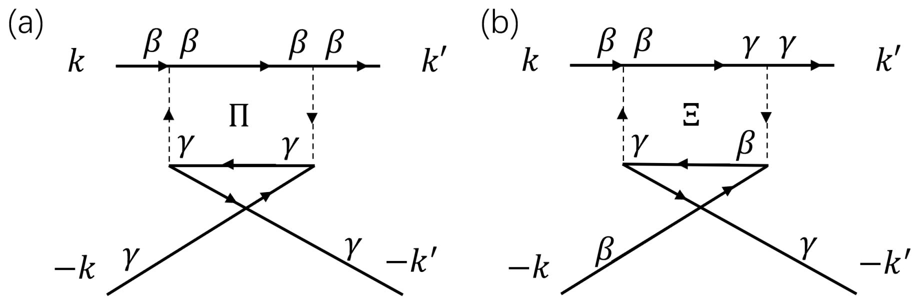

Figure S15. Pairing vertex mediated by two different bond fluctuations: Π-type (a) and Ξ-type fluctuations.

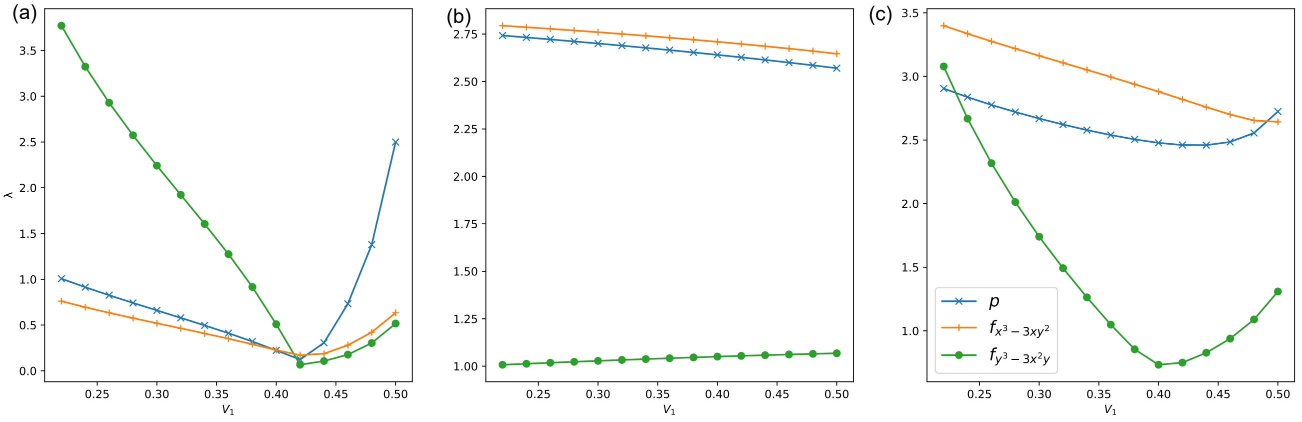

Figure S16. Leading pairing symmetry under $\mu = 0.01$ and $\beta = 150$ along the line $V_1 + V_2 = 1.15$, when considering contribution of (a) ladder diagrams (b) bubble diagrams (c) full effective interaction

X. ROLE OF BOND AND ONSITE FLUCTUATION IN DETERMINING PAIRING SYMMETRY

Dominant pairing gap is dictated by the pairing interactions. Now we focus on the Cooper pair scattering between states near VHSs, which is mainly mediated by bond fluctuations as shown in Fig.S15. Due to the unique sublattice texture on the Fermi surface, pairing scattering between different VHSs are primarily attributed to the Ξ-type fluctuation according to Fig.S15 (b). Taking the Cooper pair scattering between $\boldsymbol{k} = \mathbf{M}_2$ and $\boldsymbol{k'} = \mathbf{M}_3$ as an example, the pairing vertex is contributed by the Ξ-type fluctuation and it is given by,

$$\Gamma(\mathbf{M}_2, \mathbf{M}_3) \approx \sum_{\eta} V_\eta V_\eta \left(-f_{1,-,\eta}(\mathbf{M}_3)[\chi(\boldsymbol{q'})]^{23,1-\eta}_{32,1-\eta} f_{1,-,\eta}(\mathbf{M}_3)\right) \propto -\left((V_1)^2[\chi(\boldsymbol{q'})]^{23,1-1}_{32,1-1} + (V_2)^2[\chi(\boldsymbol{q'})]^{23,1-2}_{32,1-2}\right). \tag{S55}$$

This can be further written as $\Gamma(\mathbf{M}_2, \mathbf{M}_3) \propto -(V_{NN}^2 \Xi_{22} + V_{NNN}^2 \Xi_{88})$ using the notations in the main text. Therefore, the sign of this effective interaction is determined by the sign of the Ξ susceptibility, which is the opposite for NN and NNN bonds. In the spinless case, this interaction should vanish at time reversal invariant points. In the main text, we consider the case with slight doping and the Fermi level moves away from VHSs. We consider two representative Fermi points near $\mathbf{M}_{2,3}$, $\boldsymbol{k} = (1-\delta)\mathbf{M}_2$ and $\boldsymbol{k'} = (1-\delta)\mathbf{M}_3$ with $0 < \delta \ll 1$. So the momentum transfer in Ξ is shifted to $\boldsymbol{q'} = \boldsymbol{k} + \boldsymbol{k'} = -(1-\delta)\mathbf{M}_1$, which is located on $\mathbf{M} - \boldsymbol{\Gamma}$ line. In the antisymmetric interaction $\Gamma(\boldsymbol{k}, -\boldsymbol{k'})$, the momentum transfer in Ξ is shifted to $\boldsymbol{q} = \boldsymbol{k} - \boldsymbol{k'} = \boldsymbol{M_1} + \delta(\boldsymbol{M_3} - \boldsymbol{M_2})$, which is located on $\mathbf{M} - \boldsymbol{K}$ line. From the Fig.2 in main text, we observe that the susceptibilities decays much faster on the $\mathbf{M} - \boldsymbol{K}$ direction than the $\mathbf{M} - \boldsymbol{\Gamma}$ direction, implying $|\Gamma(\boldsymbol{k}, \boldsymbol{k'})| > |\Gamma(\boldsymbol{k}, -\boldsymbol{k'})|$. Therefore, the pairing spinless vertex is dominantly determinated by $\Gamma(\boldsymbol{k}, \boldsymbol{k'})$. With this, the NN Ξ-type bond fluctuation induces a repulsive scattering interaction but the NNN Ξ-type bond fluctuation induces an attractive between k points near $\mathbf{M}_2$ and $\mathbf{M}_3$. When V_2 is dominant, the gap function on around $\mathbf{M}_2$ and $\mathbf{M}_3$ should have the same sign and this leads to the $f_{y^3-3x^2y}$-wave pairing shown in the main text. Since the antisymmetrization of interaction cancels most of

the vertex $\Gamma(\mathbf{M}_2, \mathbf{M}_3)$, the scattering is prominent when the susceptibility is nearly divergent. This explains why the $f_{y^3-3x^2y}$-wave pairing only emerges in a small regime of the pairing phase diagram (Fig.6(b) in the main text).

Away from VHSs, espetially near the midpoint between two VHSs (P_2 in the main text), the contribution of bubble-type diagrams becomes important. This bubble-type diagram contains onsite charge susceptibility peaking at $\boldsymbol{q} = 0$. Near the $\pm P_2$ point, the contribution from bubble diagram can overcome that of ladder diagram when V_2 is dominant, as shown in Fig.5 (d),(f) in the main text, and this results a repulsive interaction between two opposite edges of the Fermi surfaces. This leads to $f_{x^3-3xy^2}$-wave pairing. When V_1 is dominant, the dominant real bond fluctuations generate an attractive interaction between two opposite edges, leading to a p-wave pairing, as shown in the main text. In Fig.S16, we plot the pairing strength from ladder-type (a), bubble-type and total contribution as function of V_1 in the $V_1 + V_2 = 1.15$ line regime. It is evident that the bond fluctuations favor the $f_{y^3-3x^2y}$-wave and p-wave pairing for the V_2-dominant and V_1-dominant regimes, respectively. In contrast, the onsite charge fluctuations favors the $f_{x^3-3xy^2}$-wave pairing. Increasing V_1 will drive a phase transition from the $f_{x^3-3xy^2}$-wave pairing to the p-wave pairing, as shown in Fig.S16 (c).

XI. SUPPRESSION OF TRIPLET SUPERCONDUCTIVITY UNDER NON-MAGNETIC IMPURITY

Since for triplet pairing symmetry, $\Delta(k) = -\Delta(-k)$, then it must be nodal to have a sign change along the Fermi surface. Small amount of non-magnetic disorder will change the superconducting gap size and critical temperature. For instance, if we consider a pairing with finite angular momentum n in the electron gas [S7].

$$H = \sum_k \epsilon_k c_k^\dagger c_k + \Delta_n \cos(n\phi) c_k^\dagger c_{-k}^\dagger + \text{H.c.} \tag{S56}$$

and small amount of onsite impurity, with onsite potential:

$$V(\mathbf{r}) = \sum_i u_0 \delta(\mathbf{r} - \mathbf{r}_i) \tag{S57}$$

where $\mathbf{r}_i$ is the location of the impurity atom. The Hamiltonian in Nambu space reads $H = \epsilon_k \tau_3 + \Delta_n cos(n\phi)\tau_1$. Then we consider this problem in the T-matrix regime:

$$G = G_0 + G_0 T G_0 \tag{S58}$$

$$T_{kk'} = \frac{V_{k''k'}}{1 - V_{kk''} \sum_k G_{0,k}} \tag{S59}$$

where $G_0 = (i\omega_n - H)^{-1}$ is the 2×2 Green's function matrix for superconductor. G is the full Green's function dressed by impurities.

For a finite n,

$$g_0 = \sum_k G_{0,k} = \frac{i\omega_n \tau_0}{\sqrt{\omega^2 + \Delta_0^2 \cos^2(n\phi)}}. \tag{S60}$$

Where τ_i is the Pauli matrix span the particle-hole space. As $\int_0^{2\pi} \cos(n\phi) d\phi = 0$, the $\Delta_n \tau_1$ term in g_0 vanishes. This is the main difference between SC with angular momentum and s-wave SC. The $\epsilon_k \tau_3$ term also vanishes for a constant DOS near the Fermi surface. When impurity scattering is weak, self-energy can be written as $\hat{\Sigma}(i\omega_n) = n_{\text{imp}} \langle \hat{T}(i\omega_n) \rangle_k$. Thus, the self-energy term can only modify the ω_n term in the Green's function.

After considering disorder, the self-consistent equation changes to:

$$\Delta_n = 2 g_n \pi T \sum_{i\omega_n} \int \frac{d\phi'}{2\pi} \frac{\Delta_n \cos^2(n\phi)}{\sqrt{\omega_n^2 (1 + \eta_{\omega_n})^2 + \Delta_n^2 \cos^2(n\phi)}} \tag{S61}$$

where

$$\eta_{\omega_n} = \frac{1}{2\Gamma} \int \frac{d\phi'}{2\pi} \frac{1 + \eta_{\omega_n}}{\sqrt{\omega_n^2 (1 + \eta_{\omega_n})^2 + (\Delta_n \cos(2\phi'))^2}}, \tag{S62}$$

with Γ being the life time of quasi-particle $\frac{1}{2\Gamma} = -\mathrm{Im}\Sigma(0)$. Since the Δ_n term enters the modification of ω_n, the structure of gap equation changes, which is different from s-wave superconductivity. Thus equation (S61) suggests that nonmagnetic impurity scattering is pairing breaking and can suppress the transition temperature as long as the Cooper pair carries non-zero angular momentum. This leads to the famous Abrikosov-Gorkov result:

$$\ln\frac{T_c}{T_{c0}} = \psi\left(\frac{1}{2}\right) - \psi\left(\frac{1}{2} + \frac{1}{4\pi\Gamma T_c}\right) \tag{S63}$$

ψ is the digamma function and T_{c0} is the transition temperature of a pure system. Then T_c decreases when increasing impurity scattering.

Since all triplet pairing symmetries are nodal, this result applies to all triplet cases in our calculation. So all obtained states in our RPA calculation will have a narrower gap and decreased transition temperature when considering non-magnetic disorder effects.

[S1] A. T. Rømer, P. J. Hirschfeld, and B. M. Andersen, Superconducting state of sr_2ruo_4 in the presence of longer-range coulomb interactions, Phys. Rev. B **104**, 064507 (2021).

[S2] D. Xiao, M.-C. Chang, and Q. Niu, Berry phase effects on electronic properties, Rev. Mod. Phys. **82**, 1959 (2010).

[S3] S. Graser, T. A. Maier, P. J. Hirschfeld, and D. J. Scalapino, Near-degeneracy of several pairing channels in multiorbital models for the fe pnictides, New Journal of Physics **11**, 025016 (2009).

[S4] A. F. Kemper, T. A. Maier, S. Graser, H.-P. Cheng, P. J. Hirschfeld, and D. J. Scalapino, Sensitivity of the superconducting state and magnetic susceptibility to key aspects of electronic structure in ferropnictides, New Journal of Physics **12**, 073030 (2010).

[S5] X. Wu, F. Yang, C. Le, H. Fan, and J. Hu, Triplet p_z-wave pairing in quasi-one-dimensional $A_2cr_3as_3$ superconductors ($a = \mathrm{K, Rb, Cs}$), Phys. Rev. B **92**, 104511 (2015).

[S6] M. Dürrnagel, J. Beyer, R. Thomale, and T. Schwemmer, Unconventional superconductivity from weak coupling, The European Physical Journal B **95**, 112 (2022).

[S7] *Bogoliubov-de Gennes Method and Its Applications* (Springer Cham).
